# Fungi promote cross-domain interactions even in deep anoxic mangrove sediments

**DOI:** 10.1186/s40793-025-00686-6

**Published:** 2025-03-25

**Authors:** Ming Sheng Ng, Nathaniel Soon, Min Yi Chin, Sze Koy Ho, Lynn Drescher, Mohamad Azlin Bin Sani, Kiah Eng Lim, Benjamin J. Wainwright, Ying Chang

**Affiliations:** 1 Department of Biological Sciences, National University of Singapore, 16 Science Drive 4, Singapore, 117558 Singapore; 2 Yale-NUS College, National University of Singapore, 16 College Avenue West, Singapore, 138527 Singapore; 3 Thrive Conservation, Jl. Subak Sari No. 13, Kuta Utara, Badung, Bali, 80361 Indonesia

**Keywords:** Fungal networks, Co-occurrence interactions, Cross-domain interactions, Fungal-bacteria interactions, Microbial stratification

## Abstract

**Background:**

Microbial communities in mangrove sediments play vital ecological roles that underpin the functioning of the overall mangrove ecosystem. Fungal communities, in particular, are known to play crucial roles across sediment systems, yet their roles in mangrove sediments, especially in deeper layers, remain poorly understood without a comprehensive inter-domain characterization. To better understand fungal roles in sediment horizons, 10 sediment cores extending down to a depth of 1 m were taken in three mangrove sites to characterise the archaeal, bacterial, and fungal communities at 10 cm depth intervals.

**Results:**

We demonstrate that sediment depth has distinct effects on the three microbial communities. While fungal community compositions were similar across sediment depths, bacterial and archaeal community compositions were stratified into three distinct layers, surface (10–30 cm), subsurface (40–60 cm), and deep (70–100 cm). Co-occurrence networks were then constructed to investigate the roles of fungi in these sediment layers, where fungi were consistently identified as keystone taxa in maintaining the microbial network topology, with co-domain interactions constituting more than half of all interactions. Even in the deepest layer, fungal nodes still retained high betweenness centralities, acting as network hubs to potentially augment microbial interactions vital for the functioning of the overall ecosystem.

**Conclusions:**

Overall, our results emphasise the important role of fungi in mediating microbial interactions across sediment depths even in deep, anoxic sediment layers, and highlight the importance of cross-domain interactions as integral to a more holistic understanding of the mangrove microbiome.

**Supplementary Information:**

The online version contains supplementary material available at 10.1186/s40793-025-00686-6.

## Background

Mangroves are highly productive ecosystems that play critical roles in coastal protection, land accretion, and the provision of habitat and nurseries for a wide array of marine and terrestrial organisms [1]. Mangrove ecosystems are also vital carbon sinks where autochthonous and allochthonous detritus is trapped and buried [2]. Contained within these sediments are diverse microbial communities that drive the processes essential for the high productivity and functioning of mangrove ecosystems [3–6].

The microbial community present in mangrove sediments comprises mainly bacteria and fungi, which constitute up to 91% of the total microbial biomass [7]. Bacteria in mangrove sediments have diverse functions such as nitrogen fixation, phosphate solubilisation, sulphate reduction, and methanogenesis, each contributing to major biogeochemical and nutrient cycling pathways [8]. Fungi, on the other hand, are fundamental decomposers and nutrient cyclers in mangrove ecosystems. Most fungi found in mangroves are saprophytes that secrete digestive enzymes to degrade biopolymers such as cellulose, lignin, and chitin [9–11]. Although archaea, viruses, protists, and algae account for a smaller component of the microbial biomass [7], their roles in mangrove sediments also cannot be understated. Archaea, in particular, are metabolically diverse and widespread in deep, anoxic sediment layers, where they contribute to fundamental processes such as ammonia and methane cycling [12, 13].

These microbial groups, however, do not function in isolation. Their interactions, particularly those involving fungi, are central to the overall structure and dynamics of the mangrove sediment microbiome [14–16]. For instance, bacteria can hitchhike on vast mycelial networks of filamentous fungi in soil to colonise new microhabitats [17]. Many fungi also harbour endohyphal bacteria that can either be mutualistic or pathogenic [14, 18, 19]. Fungi and bacteria can either compete or synergistically degrade compounds such as lignin and cellulose [20]. Fungal growth has also been observed to have a strong negative relationship with archaeal abundance likely through competitive interactions [21]. These inter-domain interactions likely change with increasing sediment depth as oxygen levels and the associated changes in redox levels within the sedimentary layers create distinct depth preferences across prokaryotic groups [22–24]. For instance, mangrove sediment bacterial communities and their associated functions are vertically stratified from methanogenesis and sulphur reduction groups to nitrogen fixers with increasing sediment depth [25–27]. Research has therefore begun to steer away from only topsoil characterisation (i.e., 0–30 cm) towards the inclusion of deeper layers for a more thorough understanding of microbial roles and functions in the sediment. While metagenomic approaches are increasingly used to comprehensively characterize co-domain microbial communities, fungal metagenomic-assembled genomes often suffer from low completeness and poor taxonomic resolution due to the complexity of the eukaryotic genome and the low fungal biomass, especially in deep sediments [28, 29]. Consequently, many studies only focus on the prokaryotic communities, neglecting the role of the mycobiome in driving cross-domain interactions across the overall microbial network.

Hence, to investigate the role of the fungal community in the overall mangrove sediment microbiome, we comprehensively characterized the interdomain microbial community in mangrove sediments at 10 cm depth intervals down to a depth of 1 m through an amplicon-based metabarcoding method targeting the prokaryotic 16S rRNA and fungal ITS regions. Alongside this, we measured the physicochemical properties of the sediment at each interval to assess potential environmental drivers in the assembly of each microbial community. To evaluate the roles of fungi in the overall mangrove sediment microbiome, we constructed co-occurrence networks down to the deep layer to identify potential keystone members that may be crucial in mediating interactions. In doing so we provide a comprehensive and fine-scale characterisation of the microbial community present in mangrove sediments to a maximum depth of 1 m. Additionally, we provide a preliminary understanding of the role of fungi in mediating cross-domain interactions that are likely essential for ecological and biogeochemical processes.

## Methods

### Sampling sites and methods

Sediment samples were collected from three mangrove sites in Pulau Ubin, Singapore: Chek Jawa (CJ), Ubin Living Lab (UL) and Jalan Durian (JD, Figure S1). At each site, a transect was laid parallel to the coastline and ten cores 10 m apart were collected with a sediment corer to obtain samples to a depth of 1 m. Along each core, approximately 30 g of sediment was collected at 10 cm intervals and stored in Longmire’s solution for DNA analysis [30]. The remaining sediment from each section was stored in Whirlpak bags and used for the analysis of sediment physicochemical properties. In total, we collected 193 samples across three sites (Table S1).

## Sediment analysis

For physicochemical characterisation, sediment samples were dried at 50 °C until a constant mass was achieved. Dried sediment samples were lightly ground with a mortar and pestle and sieved with a 2 mm sieve to remove macroflora, wood fragments, and coarse mineral fragments. A total of six parameters were measured, namely, carbon, nitrogen, sulphur, and phosphorus compositions, pH, and particle size distribution.

Sediment pH was measured with a pH combination electrode (Mettler Toledo, Switzerland) in 1:2.5 soil–water ratio extracts [31]. Sediment carbon, nitrogen, and sulphur compositions (%) were measured with a CHNS Elemental Analyser (Thermo Fisher Scientific, Waltham, MA, USA) while phosphorus composition (ppm) was measured with the Inductively Coupled Plasma-optical Emission Spectrometer (Perkin Elmer, Waltham, MA, USA). Sediment particle sizes were measured with a Partica LA-960V2 Laser Scattering Particle Size Distribution Analyzer (Horiba Scientific, Kyoto, Japan).

## DNA extraction and amplification

Extraction of DNA in homogenised sediment samples was conducted with the QIAGEN DNeasy PowerLyzer PowerSoil Kits following the manufacturer’s protocol with no modifications. The bacterial and archaeal V4 region of the 16S rRNA was amplified with the modified 515F [32] and 806R [33] primer pair, while the ITS2 region of fungal DNA was amplified with the fITS7 and ITS4 primer pair [34, 35]. Negative extraction and PCR controls were included and sequenced to identify any potential contamination. For a detailed description of the methods used, see SI methods. Fungal and prokaryotic libraries were sequenced separately on the Illumina MiSeq platform (600 cycles, V3 chemistry, 300 bp paired-end reads), both with a 30% PhiX spike by Macrogen, Inc.

## Bioinformatics and statistical analyses

The bioinformatics workflow and analyses for both the V4 and ITS2 sequenced libraries closely followed the recommendations of Pauvert [36] with the DADA2 package to gather amplicon sequence variants (ASVs) [37]. Rarefaction curves were produced to ensure sufficient sequencing depth for complete diversity recovery for each sample. The V4 dataset was separated into archaeal and bacterial datasets, and all subsequent analyses were performed on the archaeal, bacterial, and fungal data independently unless otherwise stated. Alpha-diversity indices, namely, Shannon diversity, species richness, and Simpson’s evenness were calculated. Linear mixed effect models (LME) were used to investigate whether communities showed significantly different alpha-diversity measures across sites and depths, accounting for random effects within each sediment core. Sediment depth was modelled as a continuous variable to identify general vertical sediment trends. All models were checked for normality and homoscedasticity via diagnostic plots to ensure model validity. To better meet the assumptions of homoscedasticity, bacterial richness was log-transformed while a variance structure of ‘weights = Depth^2’ was used to model bacteria evenness to account for within-group errors.

For beta-diversity, raw sequence counts were transformed to scale-invariant robust centred log-ratios to account for sparsity, compositional effects, and uneven sequencing depths [38]. Principal component analysis (PCA) was then conducted to visualise the microbial communities with Euclidean distances, equivalent to robust Aitchison distances. Permutational analysis of variance (PERMANOVA) was then conducted with Euclidean distances to investigate whether the community compositions differed across sediment depths and sampling sites, stratified within the sediment cores. We treated depth as a categorical variable to test for differences between the ten sediment layers. Since depth significantly influenced the archaeal and bacterial community compositions, pairwise comparisons were conducted with the *pairwiseAdonis* package [39], controlling for false discovery rate [40]. Post-hoc pairwise analysis revealed that bacterial and archaeal communities were stratified into three layers: 10–30 cm (surface), 40–60 cm (subsurface), and 70–100 cm (deep), where the community compositions were significantly different across the layers, but similar within each layer (Tables S5and S6). These depth groupings also maximised the between-group variations and minimized the within-group variations compared to other depth grouping combinations in the PCA.

Taxa that were differentially abundant across the three layers were identified with the *Corncob* package [41]. Analyses were conducted at the class level to retain statistical power and reduce false discovery rate. To further improve the robustness of the results, analyses were with a false discovery rate of 0.05 [40]. Classes with adjusted *p* of less than 0.05 were considered as significantly differentially abundant between layers.

To investigate whether the six sediment geochemical properties, pH, mean particle size (µm), carbon %, nitrogen %, sulfur %, and phosphorus (ppm), significantly changed with sediment depth and across sampling sites, separate LME models were used, accounting for random effects within each core. These sediment properties were then tested for significance in the assembly of the archaeal, bacterial, and fungal communities with Distance-Based Redundancy Analysis (db-RDA). Likewise, community data was first transformed to robust centred log ratios, before being expressed on the dissimilarity matrix in Euclidean space. Only sediment geochemical parameters that were significant (*p* < 0.05) were retained in the db-RDA plots.

## Network analysis

An inter-domain co-occurrence network was constructed for each layer (surface, subsurface, and deep) to examine cross-domain interactions and the roles of the fungal community. Samples across the three different sites were pooled together to construct the three networks with the SPIEC-EASI package [42]. While site-specific differences can have significant effect on cross-domain interactions, sample pooling allowed for a broader investigation of fungal roles and the identification of keystone taxa across mangrove sites. Five centrality measures were calculated to assess the roles of each node in their respective network: eigenvector (the measure of the influence of a node in a network, ranging from 0 to 1), closeness (reciprocal of the average distance from a node to all other nodes in the network), eccentricity (distance between a node and its furthest node), degree (number of edges a node has), and betweenness (number of shortest paths between any two nodes in the network passing through that node) centralities. Analysis of variance (ANOVA) was conducted to determine if centrality measures varied significantly across domains and layers, as well as their interactions. Since ‘Layer × Domain’ was significant across all models, pairwise analysis was conducted with the *emmeans* package to investigate which domain had the highest centrality within each sediment layer. Eccentricity centrality did not fit any distribution and was not analysed with ANOVA.

To further investigate the roles of each network node, within-module connectivity (Z_i_) and participation coefficient (P_i_) were calculated as defined in [43]. Briefly, Z_i_ describes a node’s connections to other nodes within its module, while P_i_ describes its participation with nodes from other modules. Modules were first calculated with the modularity optimisation algorithm described in [44] with the *igraph* package [45], and weighted Z_i_ and P_i_ were calculated with the *brainGraph* package (https://github.com/cwatson/brainGraph). Nodes were divided into four roles based on their Z_i_ and P_i_ values following [46]: (i) peripherals (Z_i_ ≤ 2.5, P_i_ ≤ 0.62) as nodes with a few links mostly within their modules, (ii) connectors (Z_i_ ≤ 2.5, P_i_ > 0.62) as nodes that are well-connected to other modules, (iii) module hubs (Z_i_ > 2.5, P_i_ ≤ 0.62) as nodes that are highly connected within their modules, and (iv) network hubs (Z_i_ > 2.5, P_i_ > 0.62) as nodes that are well-connected across modules.

## Results

### Microbial alpha-diversity along sediment depth

The filtering of rare ASVs with less than 1% prevalence and low taxonomic resolution (no ‘Class’ assignment) resulted in 1,607 archaeal ASVs, 7,342 bacterial ASVs, and 2,086 fungal ASVs across all sediment depths and sites.

**Fig. 1:**
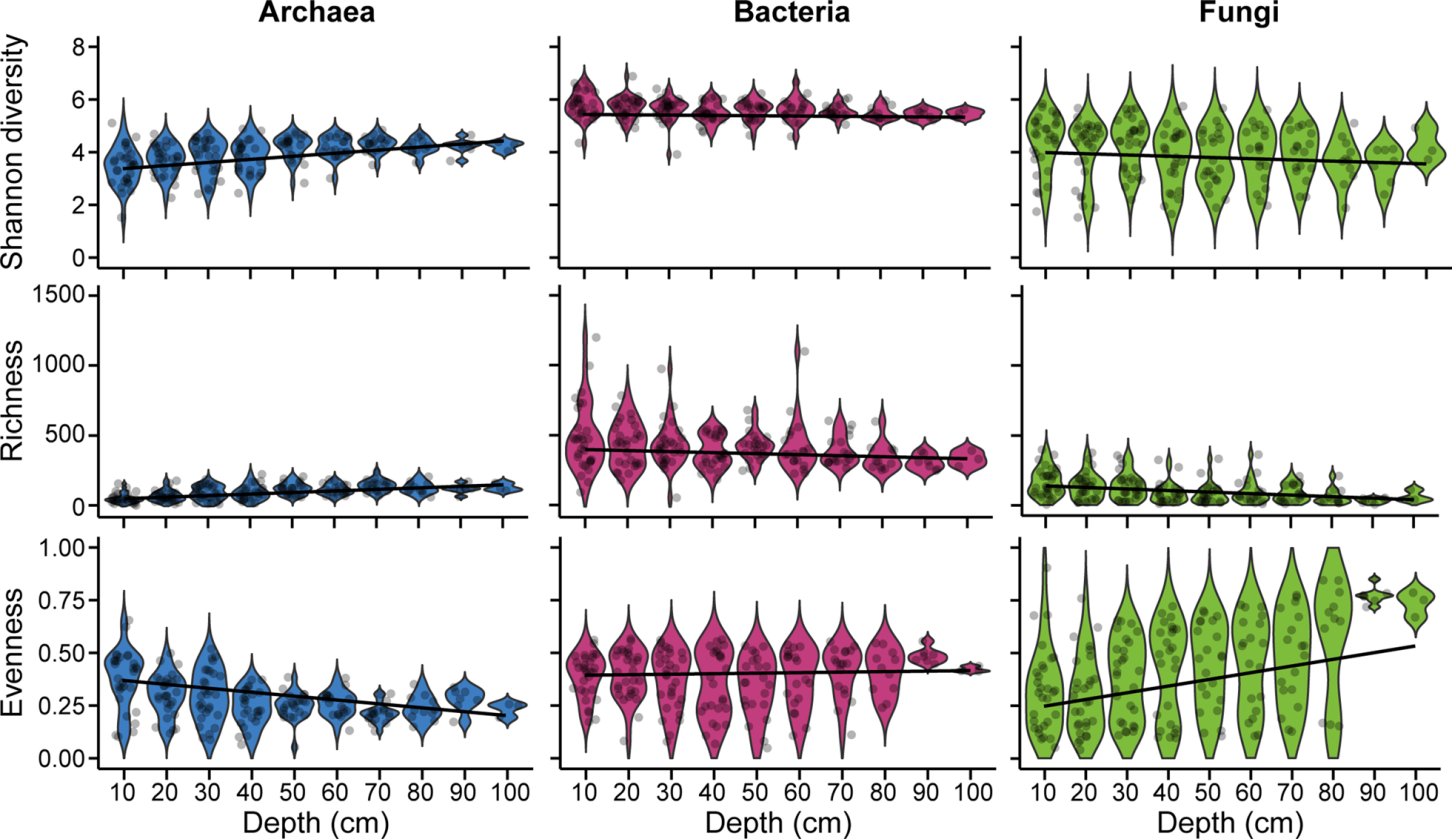
Changes in the three alpha-diversity indices, Shannon diversity, richness, and evenness, of the mangrove archaeal, bacterial, and fungal communities with sediment depth. Lines represent fits of the linear mixed effect models

As sediment depth increased from 10 to 100 cm, archaeal Shannon diversity increased significantly from 3.17 ± 0.13 (mean ± SE) to 4.15 ± 0.11 (LME, F-value = 58.8721, *p* < 0.0001; Fig. 1; Table S4), driven by a significant increase in richness from 47.8 ± 5.5 to 128.0 ± 16.3 (LME, F-value = 83.4177, *p* < 0.0001). Conversely, archaeal community evenness decreased significantly from 0.444 ± 0.026 at 10 cm depth to 0.253 ± 0.021 at 100 cm depth (LME, F-value = 64.2694, *p* < 0.0001). These indices did not significantly differ among the three mangrove sites (LME, *p* > 0.05).

In contrast, Shannon diversity, richness, and evenness of bacterial communities were similar across sediment depths and sites (LME, *p* > 0.05; Fig. 1; Table S4). Across all sites and depths, the mean Shannon diversity index was 5.40 ± 0.03, with a mean richness of 381.67 ± 9.59 and a mean evenness of 0.4064 ± 0.0093.

The Shannon diversity index of the fungal communities decreased significantly from 4.72 ± 0.20 at 10 cm depth to 4.08 ± 0.21 at 100 cm depth (LME, F-value = 25.6178, *p* = 0.0001; Fig. 1; Table S4). Fungal richness also significantly decreased from 347.0 ± 38.8 to 165.0 ± 56.1 (LME, F-value = 55.6852, *p* < 0.0001), while evenness significantly increased from 0.253 ± 0.026 to 0.679 ± 0.029 (LME, F-value = 44.106, *p* < 0.0001) as sediment depth increased from 10 to 100 cm. Sampling site also had a significant effect on all three alpha-diversity metrics, albeit to a much smaller extent than did the sediment depth (LME, *p* < 0.01, Table S4).

## Microbial beta-diversity along sediment depth

**Fig. 2:**
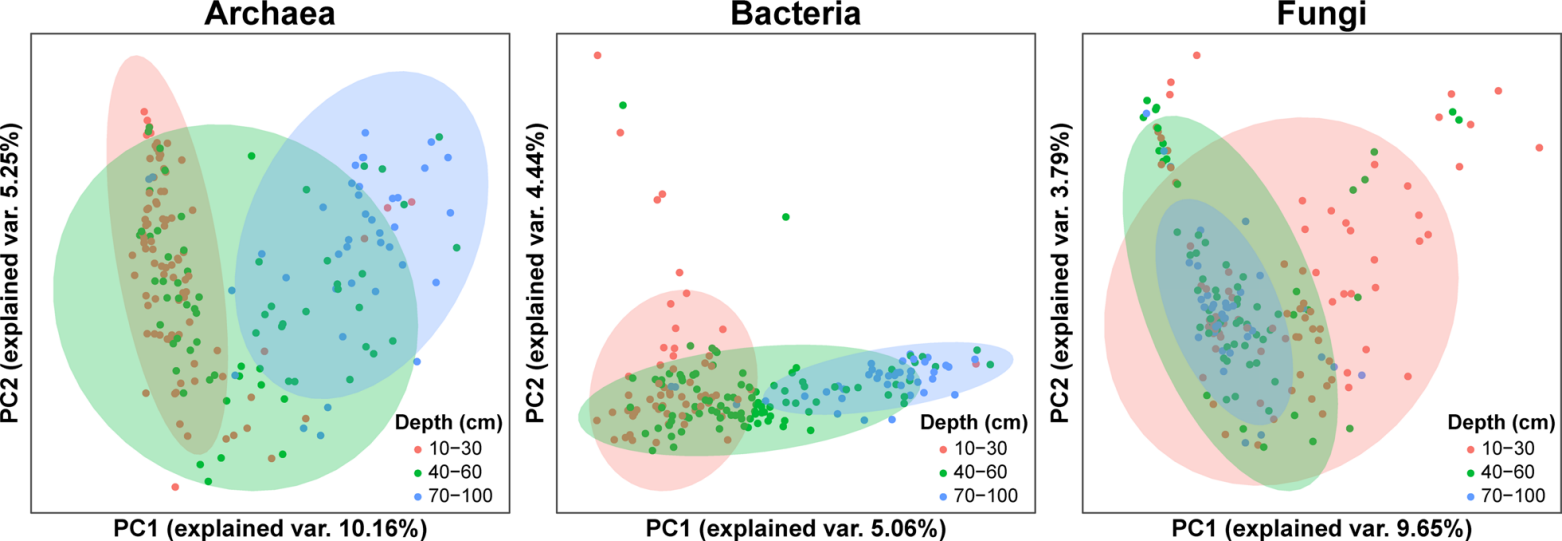
Principal component analysis plots of the archaeal, bacterial, and fungal communities found in mangrove soil. Each point represents a sample and is coloured by the depth layer – surface (10 – 30 cm), subsurface (40 – 60 cm), and deep (70 – 100 cm), while the ellipses represent 95% confidence intervals

Principal component analysis (PCA) plots were constructed to visualise archaeal, bacterial and fungal communities (Fig. 2). Both the archaeal and bacterial PCA plots showed a clear depth effect along PC1 despite substantial overlaps. While the PCA plot for fungi showed no distinct clustering with large overlaps of the surface communities with the subsurface and deep communities.

Permutational multivariate analysis of variance (PERMANOVA) emphasized that the compositions of archaeal communities were significantly different across sediment depths (F-value = 2.5186, *p* = 0.001; Table S6, Figures S – S6), while site alone did not have any significant effect (F-value = 4.1355, *p* = 0.107). Likewise, significant differences in bacterial community compositions were mostly attributed to sediment depth effects (F-value = 1.5571, *p* = 0.001), and further across sampling sites (F-value = 4.8100, *p* = 0.001). Using the criteria defined in the methods section, archaeal and bacterial communities were stratified into surface (10–30 cm), subsurface (40–60 cm), and deep (70–100 cm), where post-hoc pairwise analysis illustrated that the archaeal and bacterial community compositions were significantly different across the layers, but similar within each layer (Tables S6 and S7).

Conversely, PERMANOVA illustrated a weak but significant depth effect in structuring fungal communities (F-value = 1.0074, *p* = 0.015; Table S5, Figures S4–S6), although post-hoc pairwise analysis revealed that fungal communities were similar across all depth pairs (Table S9). Sampling sites did not have a significant effect on the composition of the fungal communities (F-value = 5.7846, *p* = 0.168).

## Identifying differentially abundant taxa across sediment layers

**Fig. 3:**
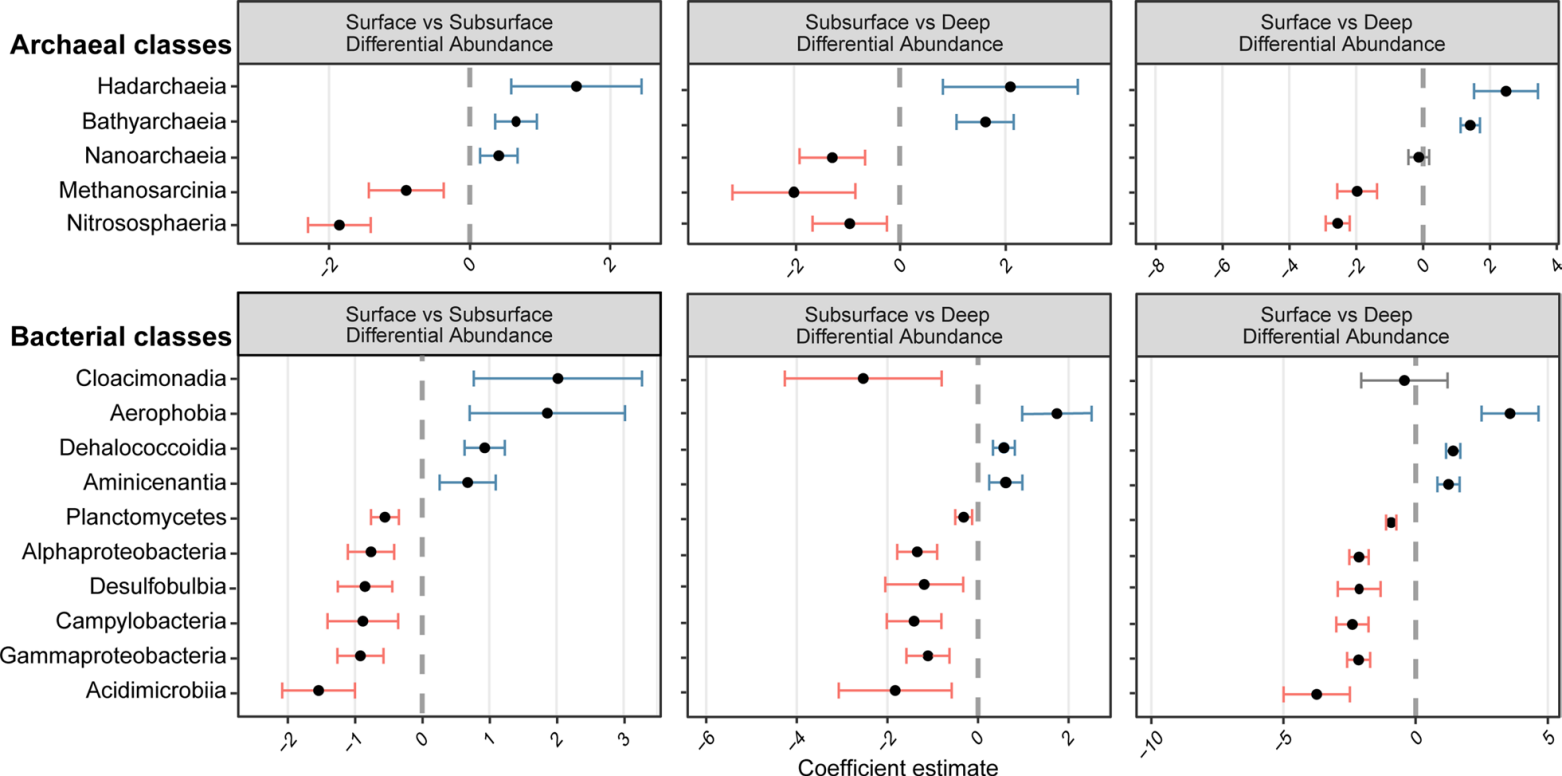
Differentially abundant bacterial and archaeal classes identified by *Corncob*. Only classes that were differentially abundant between the surface and subsurface layers and between the subsurface and deep layers were highlighted here. The blue lines refer to taxa with significantly increased abundance, the red lines refer to taxa with significantly decreased abundance, while the grey lines refer to taxa with no differential abundance compared to the baseline. There were no differentially abundant fungal classes identified across the three different sediment layers

Differentially abundant classes across the three sediment layers, surface, subsurface, and deep, were identified by *Corncob* analysis (Figs. 3 and S3). In particular, classes that were differentially abundant between the surface and subsurface layers and between the subsurface and deep layers were highlighted. The results from *Corncob* corroborated PERMANOVA and PCA results with no differentially abundant fungal classes across the three sediment layers. Since sediment depth was the main explanatory variable for archaeal and bacterial communities, we focused the analysis on the sediment layers rather than sampling sites.

The surface layer harboured more archaeal classes *Methanosarcinia* and *Nitrososphaeria*, while the deep layer had more archaea belonging to *Hadarchaeia* and *Bathyarchaeia*. In contrast, the subsurface layer had the highest abundance of archaea from the class *Nanoarchaeia*.

In the surface layers, classes with many aerobic or obligate aerobic bacteria such as *Planctomycetes* were more abundant. Whereas, taxa belonging to anaerobic classes such as *Aerophobia* and *Dehalococcoidia* were more abundant in the subsurface and deep layers.

## Relationship between sediment physicochemical properties and microbial communities

Linear mixed-effects models highlighted that sediment pH had the strongest negative relationship with sediment depth (*t* = -8.821, *p* < 0.0001), followed by phosphorus (*t* = − 5.7754, *p* < 0.0001), and mean particle size (*t* = − 5.363, *p* < 0.0001). Sulphur % showed a strong positive relationship with sediment depth (*t* = 5.804, *p* < 0.0001), while carbon had a weaker but still positive relationship (*t* = 2.920, *p* = 0.0029). Only nitrogen % did not differ significantly with sediment depth (*t* = − 1.378, *p* = 0.224; Table S10).

**Fig. 4:**
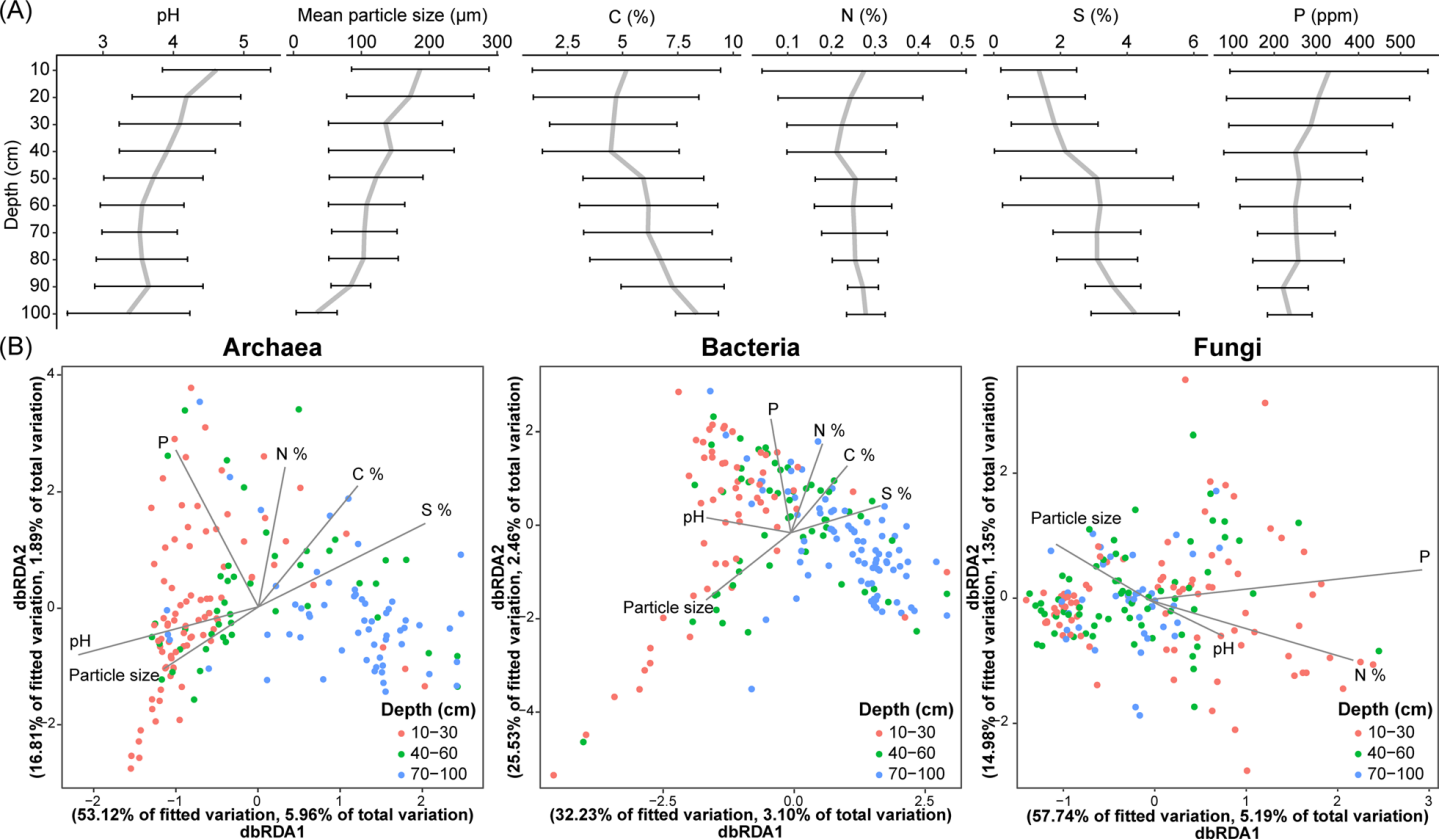
**A** Changes in the six analysed environmental parameters with mangrove sediment depth and **B** Distance-based redundancy analysis (db-RDA) of archaeal, bacterial, and fungal communities illustrating the parameters significant in explaining their assemblies

We then investigated how these measured sediment properties correlate with the microbial community composition. Distance-based redundancy analysis (db-RDA) indicated that bacterial and archaeal beta diversities were significantly correlated with pH, particle size, phosphorus, nitrogen %, sulphur %, and carbon % (Fig. 4; Tables S11 and S12). The db-RDA1 for both the archaeal and bacterial communities were positively loaded with sulphur % and carbon %, and negatively loaded with pH and particle size. Meanwhile, sediment particle size, phosphorus, nitrogen % and pH were significantly correlated with fungal beta diversity (Table S13), where its db-RDA1 was instead strongly and positively loaded with phosphorus and nitrogen %.

## Cross-domain network and centrality measures

**Fig. 5:**
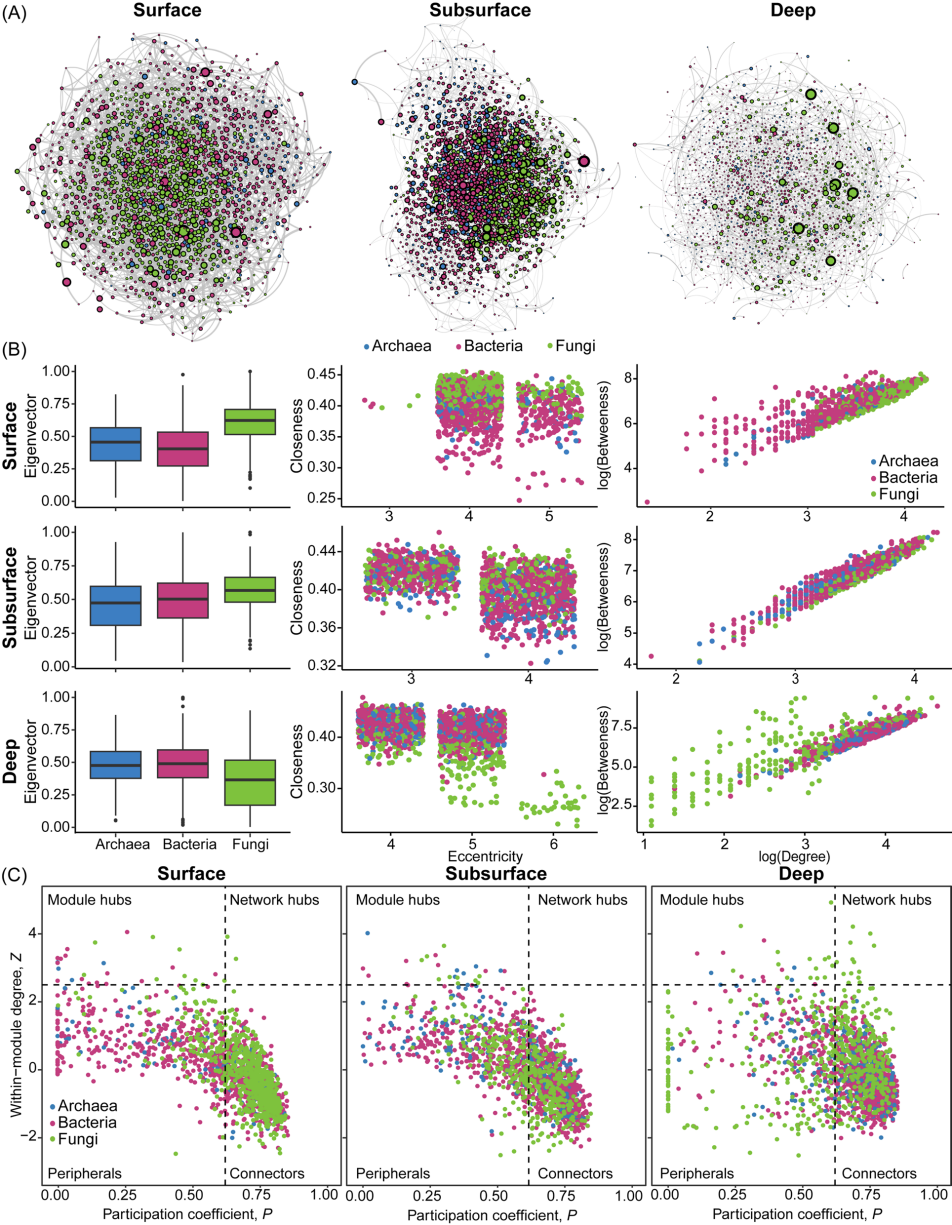
**A** Cross-domain networks for the surface, subsurface, and deep microbial communities. The size of the nodes is scaled to their betweenness centrality. **B** Keystone species analysis of all ASVs in the surface, subsurface, and deep cross-domain co-occurrence network: Eigenvector centrality, closeness centrality vs eccentricity, log(Betweenness centrality) vs log(Degree). **C** Within-module connectivity and participation coefficient of each node in each network

Cross-domain networks were constructed for the surface (10–30 cm), subsurface (40–60 cm), and deep (70–100 cm) layers (Fig. 5; Table S14). Notably, the deep network had the greatest number of nodes and edges but had the lowest average degree, network density, and clustering coefficient. On the other hand, the surface network had the highest average degree and shared a similar network density and clustering coefficient as the subsurface network.

Cross-domain interactions accounted for a large proportion of the edges for all three constructed networks. The proportion of cross-domain interactions also increased with increasing depth, from 42.07% on the surface to 51.23% in the deep. In the surface and subsurface layers, these cross-domain interactions were dominated by fungi-bacteria interactions, comprising 30.09% and 31.29% respectively of the edges in the respective network. In the deep layer, fungi-bacteria interactions diminished slightly to represent 20.65% of overall interactions, while archaea-bacteria interactions dominated the network at 24.16% of all edges (Table S15).

The four network centrality measures, namely eigenvector, closeness, betweenness, and degree centrality, were significantly different across domains within each sediment layer (ANOVA, Domain × Layer, all *p* < 0.001, Table S16). Fungal nodes had the highest eigenvector, closeness, and degree centrality measures in the surface and subsurface layers compared to archaeal and bacterial nodes, but were the lowest in the deep layer (Least-Squares Means, all *p* < 0.001, Table S17). Whereas, fungal nodes had the highest betweenness centrality compared to bacterial and archaeal nodes in the surface and subsurface layers, and also in the deep layer (Least-Squares Means, *p* < 0.001). Bacterial and archaeal nodes had mostly similar centrality measures across all layers (Least-Squares Means, *p* > 0.05), except at the subsurface layer where bacterial nodes had slightly greater closeness and eigenvector centrality values (Least-Squares Means, *p* < 0.05). Overall, fungal nodes had among the highest degree, betweenness, and eigenvector centralities, forming the majority of the potentially keystone nodes especially in the surface and subsurface sediment layers.

In the surface layer, the largest keystone node was from the fungal class *Eurotiomycetes* followed by the fungal keystone node from the family *Herpotrichiellaceae*. The largest keystone node in the subsurface layer was also fungal from the genus *Trichoderma*, followed by the bacterial node from the taxon Napoli-4B-65. Instead, the top two keystone nodes in the deep layer were of bacterial origin, BD2-11 terrestrial group and Anerolineae (Table 1).

**Table 1:** Keystone nodes with the highest eigenvector were identified from the surface, subsurface, and deep sediment microbial cross-domain networks, along with their betweenness and degree centralities

Layer	ASV	Domain	Lowest taxa	Eigenvector	Betweenness	Degree
Surface	1422	Fungi	*Eurotiomycetes*	1	2272	70
	2547	Fungi	*Herpotrichiellaceae*	0.9958	2313	71
	1838	Bacteria	*Sandaracinaceae*	0.9757	2308	71
	492	Fungi	*Peroneutypa polysporae*	0.9546	1912	67
	1061	Fungi	*Aculeata*	0.9320	1762	67
Subsurface	765	Fungi	*Trichoderma*	1	2249	59
	220	Bacteria	*Napoli-4B-65*	0.9996	2239	66
	920	Fungi	*Phaeophleospora hymenocallidicola*	0.9834	2527	63
	6604	Bacteria	*Campylobacterales*	0.9516	2776	64
	6105	Archaea	*Odinarchaeia*	0.9276	2130	61
Deep	362	Bacteria	*BD2-11 terrestrial group*	1	4402	104
	1695	Bacteria	*Anaerolineae*	0.9892	3125	89
	3067	Bacteria	*Dehalococcoidia*	0.9308	2735	85
	299	Fungi	*Sordariomycetes*	0.9000	4697	85
	5479	Fungi	*Talaromyces*	0.8814	4293	81

Nodes were also classified into four potential ecological roles based on their within-module connectivity (Z_i_) and participation coefficient (P_i_) [43]. Notably, network hubs were detected only in the surface and deep networks, with two in the surface and 13 in the deep, all of which were fungal (Fig. 5; Tables S18 and S19). Most of the nodes in the three networks were classified as connectors, followed by peripherals and then module hubs.

## Discussion

Fungal communities have been shown to underpin microbial interactions by playing critical roles in mediating cross-domain interactions in complex microbiomes, such as mangrove sediments, that are paramount for the functioning of the overall ecosystem [47]. In this study, we first characterized the archaeal, bacterial, and fungal communities at 10 cm intervals to a depth of 1 m across three mangroves in Singapore. We demonstrated the distinct influences of sediment depth on the alpha and beta diversities of each microbial community. Notably, bacterial and archaeal communities were stratified into three sediment layers, surface (10–30 cm), subsurface (40–60 cm), and deep (70–100 cm), while fungal community composition remained relatively consistent across the sediment depths. Construction of co-occurrence networks then revealed the prominent role of fungi in mediating cross-domain interactions, suggesting their potential to augment the interactions of microbial groups across the entire sediment microbial network. Our results provide novel insights into the keystone role of fungi even in the deep, anoxic layers of mangrove sediments, and reinforce the importance of recognising the mangrove microbiome as a single multi-domain community with dense cross-domain interactions that are likely integral for its overall functioning.

Mangrove archaeal communities were more speciose and diverse at deeper sediment depths, with *Bathyarchaeia* dominating the communities. These archaea are ubiquitous and among the most abundant microorganisms on Earth [48], particularly in anoxic subsurface environments [49], where they play vital roles in carbon cycling through glycolysis, gluconeogenesis, and the Wood-Ljungdahl pathway, and may also metabolize complex organic compounds such as lignin and xylan [50]. Deep archaeal communities were also enriched in *Hadarchaeia* (formerly *Hadesarchaea*), which are predicted to play important roles in mediating ammonia and carbon cycling [51]. Many archaeal members are highly specialised and thrive in extreme energy-limiting and anoxic environments [51, 52], which likely accounts for them being the only microbial group with an observed increase in richness and diversity in the deep layer in this study. Coupled with the shift towards *Bathyarchaeia* and *Hadarchaeia*, this further supports the role of archaeal communities in driving biogeochemical cycles, especially in deeper, anoxic mangrove sediments [53].

In contrast, the alpha-diversity indices of the bacterial communities showed no clear variations across sediment depths or sites. Despite this, bacterial ASVs were markedly more abundant than archaeal and fungal ASVs across all depths, underscoring their dominance in the overall sediment microbial community [54]. Apart from predominantly aerobic classes such as *Alphaproteobacteria* and *Gammaproteobacteria*, surface communities were also enriched in chemolithoautotrophic bacteria such as *Campylobacteria* (mostly from the genus *Sulfurovum*) and *Desulfobulbia*, which can oxidise [55–57] or reduce various forms of sulphur essential for the sulphur cycling process [58]. Conversely, deep bacterial communities enriched with anaerobic groups such as *Aerophobia* and *Aminicenantia* also had more *Dehalococcoidia* bacteria, capable of breaking recalcitrant organochlorines down into compounds to be metabolised by other microorganisms, driving halogen cycling and potentially influencing carbon turnover and nutrient cycling [59]. A previous comprehensive metagenomic investigation of mangrove sediment highlighted a vertical stratification of genes and pathways from denitrification and sulphur reduction on the surface (0–5 cm) to methanogenesis in the deep (80–100 cm) [25]. While metabarcoding limits functional interpretation, the observed stratification of archaeal and bacterial communities also supports such a gradient of ecological role, from sulphur cycling at the surface to halogen cycling through reductive dehalogenation in the deeper layers.

Fungal communities showed a marked decline in richness but an increase in evenness with increasing sediment depth, resulting in an overall reduction in Shannon diversity. Yet, there were no discernible changes in community composition, indicating that only low-abundance and rare fungal taxa were lost with increasing sediment depth. The most prevalent fungal classes identified here were *Dothideomycetes* and *Sordariomycetes* from *Ascomycota*, aligning with other metabarcoding and culturomics investigations on mangrove sediments [60–62]. Within these classes, fungal orders *Pleosporales* and *Hypocreales* were among the most abundant, with *Pleosporales* largely comprising saprophytic fungi and potentially contributing as decomposers within mangrove ecosystems [65]. Interestingly, most of the fungal ASVs found in the anoxic subsurface and deep layers were previously known as aerobic fungi typically found in terrestrial ecosystems. These findings parallel the growing evidence of rich fungal diversity and metabolic activities in anoxic environments. For instance, the presence of fungal hyphae was confirmed in anoxic mangrove sediments [66]. Fungi isolated from anoxic sediments can also grow, form mycelia, and remain metabolically active under both aerobic and anaerobic conditions as facultative anaerobes [67–70]. Metatranscriptomic analyses further revealed that fungi can continue to decompose complex organic matter such as lignin and polysaccharides in anoxic marine sediments [71–73]. Consistent with these studies, the persistent community composition of fungal saprobes from aerobic to anaerobic layers observed here, many of which associated with decaying wood, suggests their capacity to maintain metabolically active to drive decomposition even in the deep mangrove sediments. This highlights their crucial roles in organic matter recycling, contributing to the high productivity of mangrove coastal ecosystems.

In addition to sediment depth, microbial communities were further influenced by various sediment properties. Archaeal and bacterial communities were strongly structured by pH and sulphur composition. Coupled with the enrichment of bacterial members involved in sulphur cycling on the surface and subsurface, this association with sulphur further supports the involvement of the bacterial communities in sulphur cycling. Previous studies have demonstrated that pH is a major factor in shaping archaeal and bacterial communities [74, 75], with prokaryotic groups adapting to different pH conditions through modifications in their plasma membranes [76]. Although redox levels were not measured due to the difficulties in *in-situ* measurements [77], it is reasonable to suggest that a reduction in oxygen availability with increasing depth led to a more reduced environment, where oxidation becomes limited and reduction processes dominate. This can then cascade into changes in sediment geochemistry such as pH and sulphur availability, and in turn, microbial abundance and composition [78] to account for the observed stratification of archaeal and bacterial communities. Sediment carbon and phosphorus significantly influenced the structure of the prokaryotic communities, likely due to their roles in polyphosphate and carbon degradation, further exemplifying the importance of these microbes in driving biogeochemical cycles [6]. In contrast, fungal community structure was most influenced by the sediment nitrogen and phosphorus levels. In nutrient-deficient mangrove ecosystems, both nitrogen and phosphorus are key limiting factors in microbial decomposition, with high N:P ratios promoting fungal decomposition [81]. Many fungi found in anoxic environments rely on nitrogen transformation processes such as anaerobic nitrification and denitrification to thrive in nutrient-deficient environments, playing crucial roles in regulating the availability and forms of nitrogen [67]. Nitrogen was also the only measured parameter without a significant relationship with sediment depth, corroborating the lack of structuring effect depth has on fungal communities. Meanwhile, pH had a minor role in influencing fungal community composition as they can tolerate large pH ranges by regulating their internal pH levels through cellular ion transport via the Rim101 pathway [69, 79].

The role of fungal communities across the different mangrove sediment layers was further explored through co-occurrence network analysis. Notably, fungal nodes consistently emerged as keystone players in the maintenance of the network topology especially in the surface and subsurface layers by being connected to the greatest number of nodes and facilitating connections due to their proximity to many other nodes within the networks. Previous metabarcoding investigations have identified fungi as important players in cross-domain microbial networks down to 21 cm in mangrove sediments [47]. Here, we show that this central role persists beyond 21 cm down to 1 m deep where they still maintained disproportionately large betweenness centralities by bridging connections between distant nodes despite the comparatively lower degree and eigenvector centralities. This is further reinforced by the thirteen network hubs, or nodes with the most links to other modules across the network, identified in the deep network where they were all fungal. Moreover, cross-domain interactions were integral in upholding the overall network topology, constituting approximately half of the interactions in all three networks where positive, fungi-bacteria interactions were the most prevalent. In fact, although the deep network was the sparsest, it had the greatest number of nodes, edges, and cross-domain interactions. This strongly suggests that cross-domain microbial interactions are pivotal in sediment ecosystem functioning, including deep sediment layers [18, 80], with fungi likely playing a central role in driving such interactions. Aggregations between fungi and bacteria have been observed across various environments and oxic conditions, from rhizosphere soil to anoxic granite fractures [81, 82]. Fungal hyphal networks in anoxic environments can act as superhighways for other microbial groups, particularly bacteria, to cross nutrient-poor areas and access distant nutrient resources [83–85]. Fungi also produce quorum-sensing molecules such as farnesol and tyrosol for interdomain cell–cell signalling [86], or anaerobically decompose organic compounds crucial for carbon recycling and microbial growth [87, 88]. For instance, *Exophiala oligosperma* (ASV884)*,* is an acidotolerant fungus and was identified as a deep network hub with disproportionately high betweenness. They are nitrile-hydrolysing and can produce a wide range of secondary metabolites and degrade hydrocarbons and esters [89–92], which may then be used by other microbes for growth. Several other fungal nodes identified as network hubs and/or had very high betweenness also represent unidentified isolates as indicated by BLAST (e.g., ASV208, ASV850, and ASV914), highlighting the paucity of well-understood ecological roles and functions of fungi in the overall mangrove sediment microbiome. While the exact nature of such interactions cannot be determined by our amplicon-based method, the corroboration of all the metrics gathered from the co-occurrence analyses strongly suggests the crucial role of fungi even in the deeper, anoxic layers.

Apart from fungal communities, co-occurrence networks also shed light on the importance of archaeal communities in deeper sediments due to the almost sevenfold increase in archaeal interactions compared to the subsurface layer. Coupled with the observed increase in richness and diversity, this reiterates the heightened activity of archaea in deep, anoxic sediment environments, possibly in mediating biogeochemical processes [93]. Overall, bacterial interactions were dominant in all three networks, which may be attributed to the speciose and highly diverse bacterial communities found in mangrove sediment. As the most abundant microbial group, bacteria contribute extensively to a wide array of ecological functions such as carbon degradation, denitrification, sulphite reduction, and polyphosphate degradation [6]. The pervasive influence of bacteria across the networks not only underscores their multifaceted ecological roles across all mangrove sediment depths, but also further emphasises the role of fungi in facilitating interactions for bacteria to carry out crucial ecological roles. Ultimately, the increase in co-domain interactions with increasing depth highlights the importance of recognising different microbial groups as a singular, holistic mangrove microbial community and characterising the mangrove sediment microbiome to deeper layers. Future characterisation efforts could also look towards incorporating other groups such as viruses which have been shown to have significant influences on local bacterial communities [94].

## Conclusion

This study provides crucial evidence for the central role of fungal communities in the overall mangrove sediment microbial communities through mediating cross-domain interactions even in deep anoxic layers. Through co-occurrence network analysis, our study provides a roadmap to identify potential keystone fungal members in mangrove sediments and other microbial networks. We encourage further mechanistic investigations on these identified putative fungal keystone members to provide stronger evidence for such interactions and their roles in the microbiome. Overall, the prevalence of co-domain interactions across sediment networks even to the deep layers underscores the importance of characterising different microbial groups as a singular, holistic mangrove microbial community to better understand their interactions, roles, and functions. These findings increase our understanding of mangrove microbial communities and their assembly to the deeper layers of this important ecosystem.

## Supplementary Information

Additional file 1.

## Data Availability

The dataset supporting the conclusions of this article is available in the National Centre for Biotechnology Information (NCBI) repository, under BioProject ID: PRJNA1120123. The R code used for the analyses has been deposited in https://github.com/mingshengg/mangrove_microbiome.
