# Supplementary material for: Fungi promote cross-domain interactions even in deep anoxic mangrove sediments
Source: Environ Microbiome. 2025 Mar 25;20:34. doi: 10.1186/s40793-025-00686-6 (PMC11934577; doi:10.1186/s40793-025-00686-6)
Supplement: Supplementary file 1 — Additional file 1. [file 40793_2025_686_MOESM1_ESM.docx]

**Supplementary Information**

**Amplification and sequencing**

The PCR cycling conditions followed that of [1]. Briefly, cycling began with an initial denaturation step at 94 °C for 3 min, followed by 35 cycles of 94 °C for 45 s, 50 °C for 60 s, and 72 °C for 90 s, with a final extension at 72 °C for 10 min. Each reaction was performed in a total volume of 25 µL, containing 12.5 µL of KAPA PCR buffer, 0.1 µL of KAPA 3G Enzyme (Kapa Biosystems, Inc, Wilmington, MA, USA), 0.75 µL of each primer at 10 µM, 2 µL of undiluted template, 1.5 µL of BSA, 0.5 µL of MgCl_2_, and nuclease-free water to 25 µL.

Amplification of the ITS2 region of fungal DNA was conducted using the fITS7 and ITS4 primer pair [2, 3], modified to include unique barcodes and Illumina adapters [4]. Each reaction was performed in a total volume of 25 µL using the same reagents and volumes described above. PCR cycling conditions began with an initial denaturation step at 95 °C for 3 min, followed by 30 cycles of 95 °C for 20 s, 53 °C for 15 s, and 72 °C for 20 s, with a final extension at 72 °C for 1 min. Negative extraction and PCR controls were included and sequenced to identify any potential contamination. Bacterial (ZymoBIOMICS Microbial Community DNA Standard, Zymo Research, Irvine, CA, USA) and fungal (Mycobiome Genomic DNA Mix, ATCC, Virginia, USA) mock communities were included to assess any bias in DNA extraction, PCR, and sequencing.

All PCR products were visualised on a 1% TAE buffer agarose gel to confirm amplification, then cleaned and normalised using SequalPrep^TM^ normalisation plates (Invitrogen, Frederick, MD, USA). Amplified ITS2 and V4 samples were sequenced independently along with their respective controls and mock communities on the Illumina MiSeq platform (600 cycles, V3 chemistry, 300 bp paired-end reads), both with a 30% PhiX spike by Macrogen, Inc.

**Bioinformatics analysis**

The R package DADA2 [5] was used to infer exact amplicon sequence variants (ASVs). The V4 forward reads were truncated at 250 bp and the reverse at 150 bp while the ITS2 forward and reverse reads were truncated at 270 bp and 200 bp respectively. The LULU curation algorithm [6] was used to collapse erroneous ASVs into their parent ASVs, before taxonomic assignment using CONSTAXv2 [7] with Python 3. The R package *decontam* (v1.16.0) was then used to identify and remove any contaminants DNA sequences via the prevalence-based identification method [8].

**Microbial compositions**

No archaeal ASV was found throughout all 193 samples. Bathyarchaeia dominated the archaeal communities especially at the deeper depths, while Thermoplasmata had the highest relative abundance at the shallower layers. A Bathyarchaeia ASV found in 80.83% of the samples (156 of 193 samples) with a mean relative abundance of 6.09%, and three more ASVs found in more than 65% of the samples. An ASV from the class Lokiarchaeia was also found across 77.20% of the samples (149 of 193 samples) at a mean relative abundance of 4.33%.

Likewise, no bacterial ASV was found across all 193 samples, although 11 ASVs found at a high prevalence of at least 65% (> 125 of 193 samples). Of note, an ASV from the genus *Caldithrix* was detected in 81.86% of the samples (158 of 193 samples) at a mean relative abundance of 0.63%, while *Desulfosarcina variabilis* was found in 74.09% of the samples (143 of 193 samples) at a mean relative abundance of 0.40%.

Similarly, no fungal ASVs were found across all 193 samples. An ASV from the Aspergillaceae family had the highest prevalence of 65.63% (126 of 192 samples) with a mean relative abundance of 2.40% ± 0.28%.

**Sequencing results**

In total, sequencing yielded 17,637,157 16S rRNA and 5,798,557 ITS quality-filtered reads. After the removal of chimaeras and low-quality reads, 7,137,817 16S rRNA and 3,949,379 ITS sequences were retained for analysis (Supplementary Table S2). Rarefaction curves showed that sufficient sequencing depth was attained to recover the diversity of archaeal and bacterial communities, but one sample (UL7_30) was discarded from the fungal dataset due to low sequencing depth (Supplementary Figure S2). No contaminants were detected by *decontam* for either the 16S rRNA or ITS datasets.


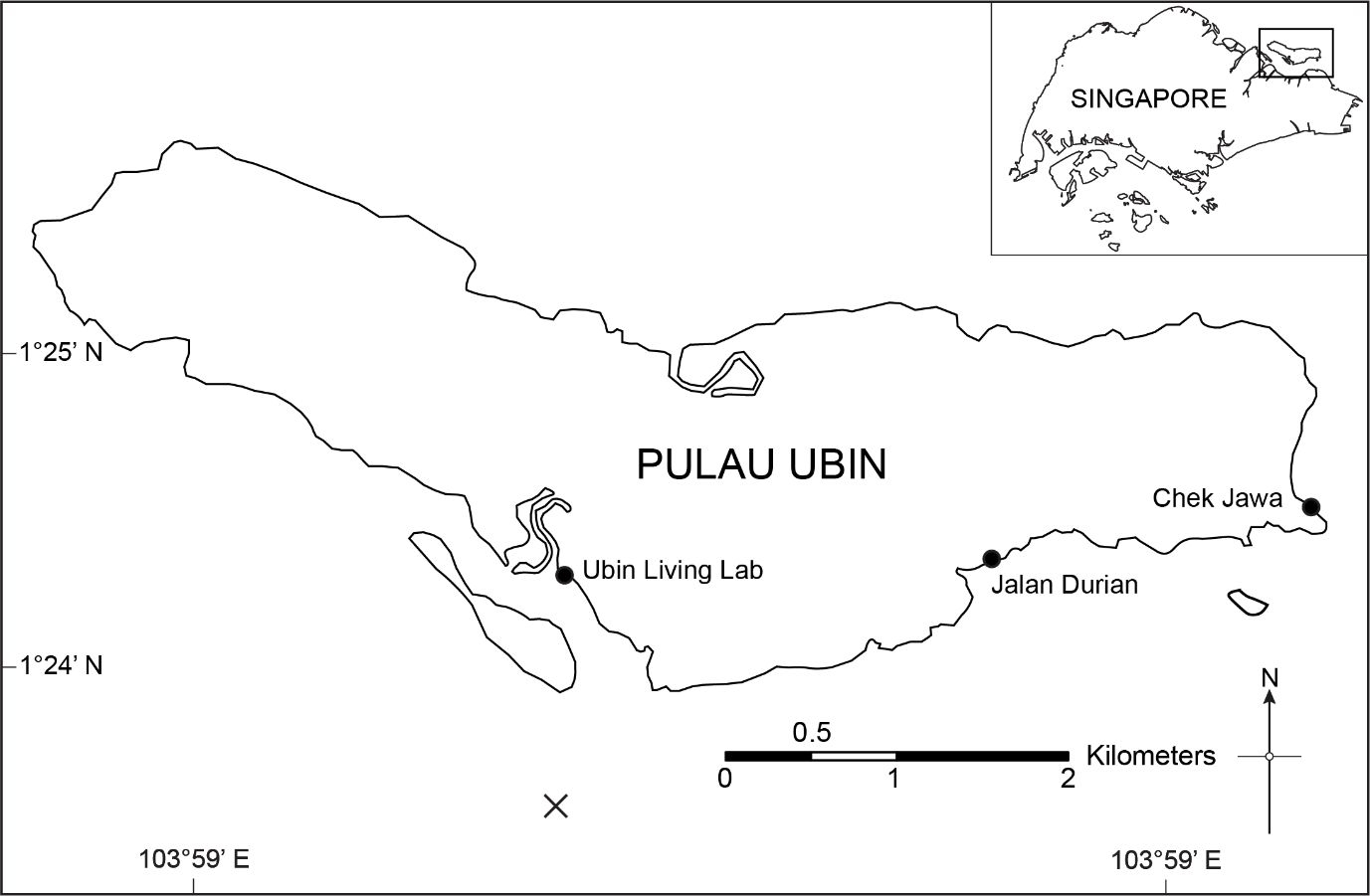


Figure S1. The three mangrove sites, Ubin Living Lab, Jalan Durian, and Chek Jawa in Pulau Ubin of Singapore. The inset depicts the Singapore map, with the box highlighting Pulau Ubin shown in the larger map.


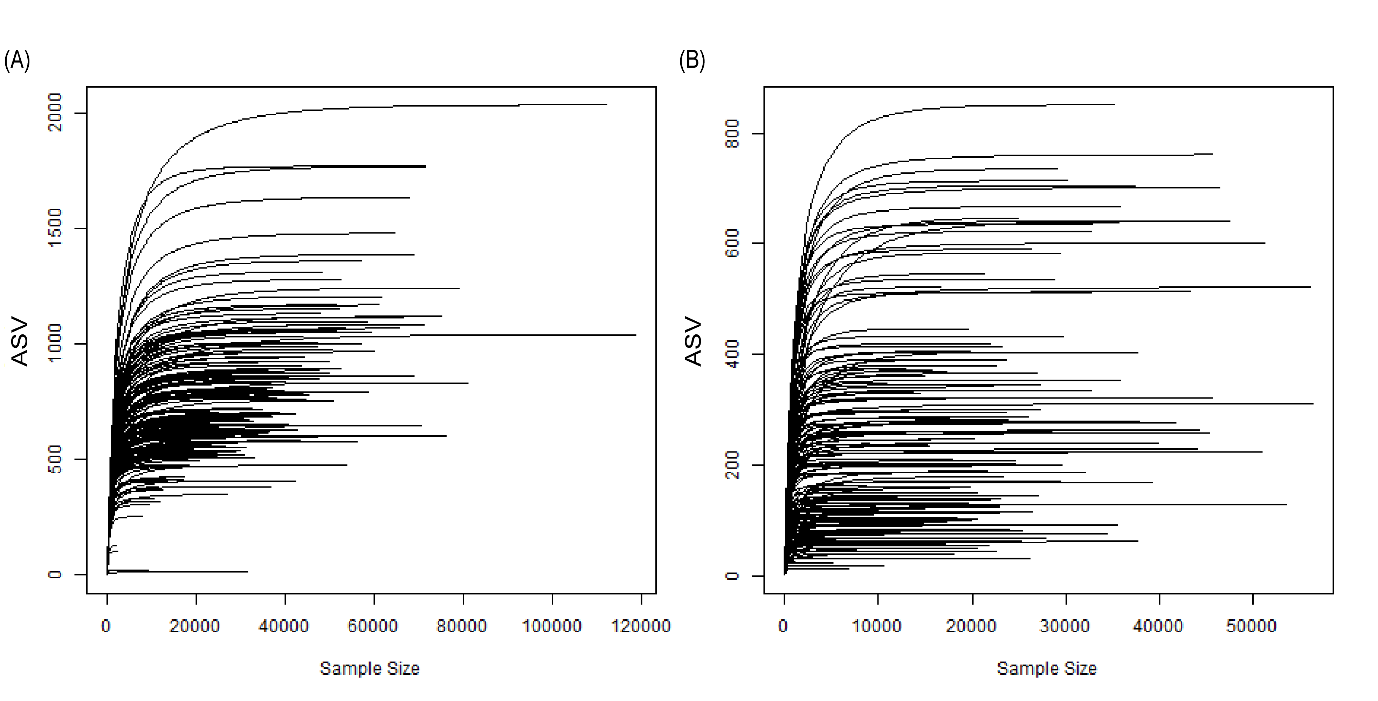


Figure S2. Rarefaction curves for (A) 16S and (B) ITS reads indicate sufficient sequencing depth to recover diversity in all samples.


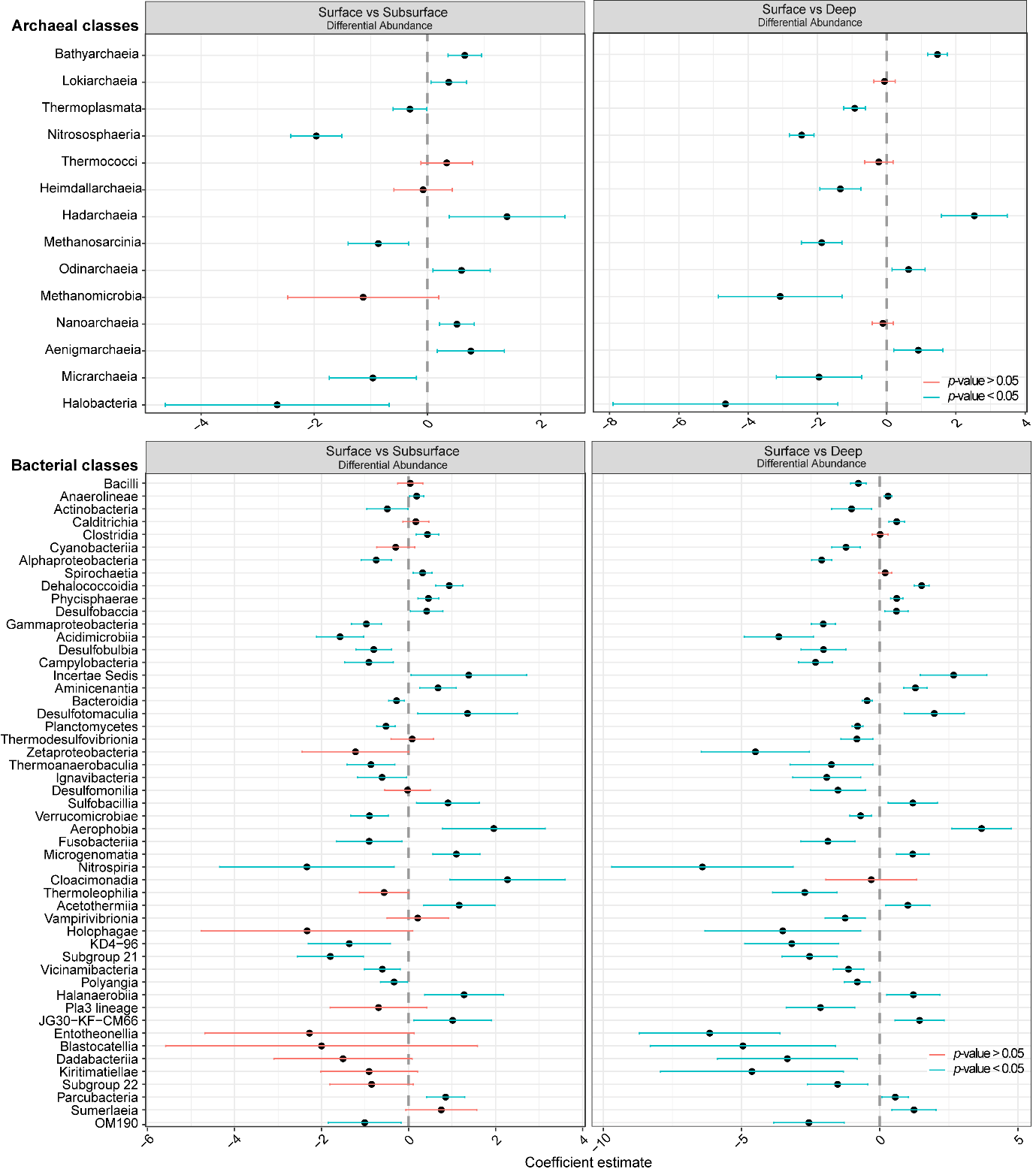


Figure S3. All differentially abundant bacterial and archaeal classes identified by Corncob analysis.


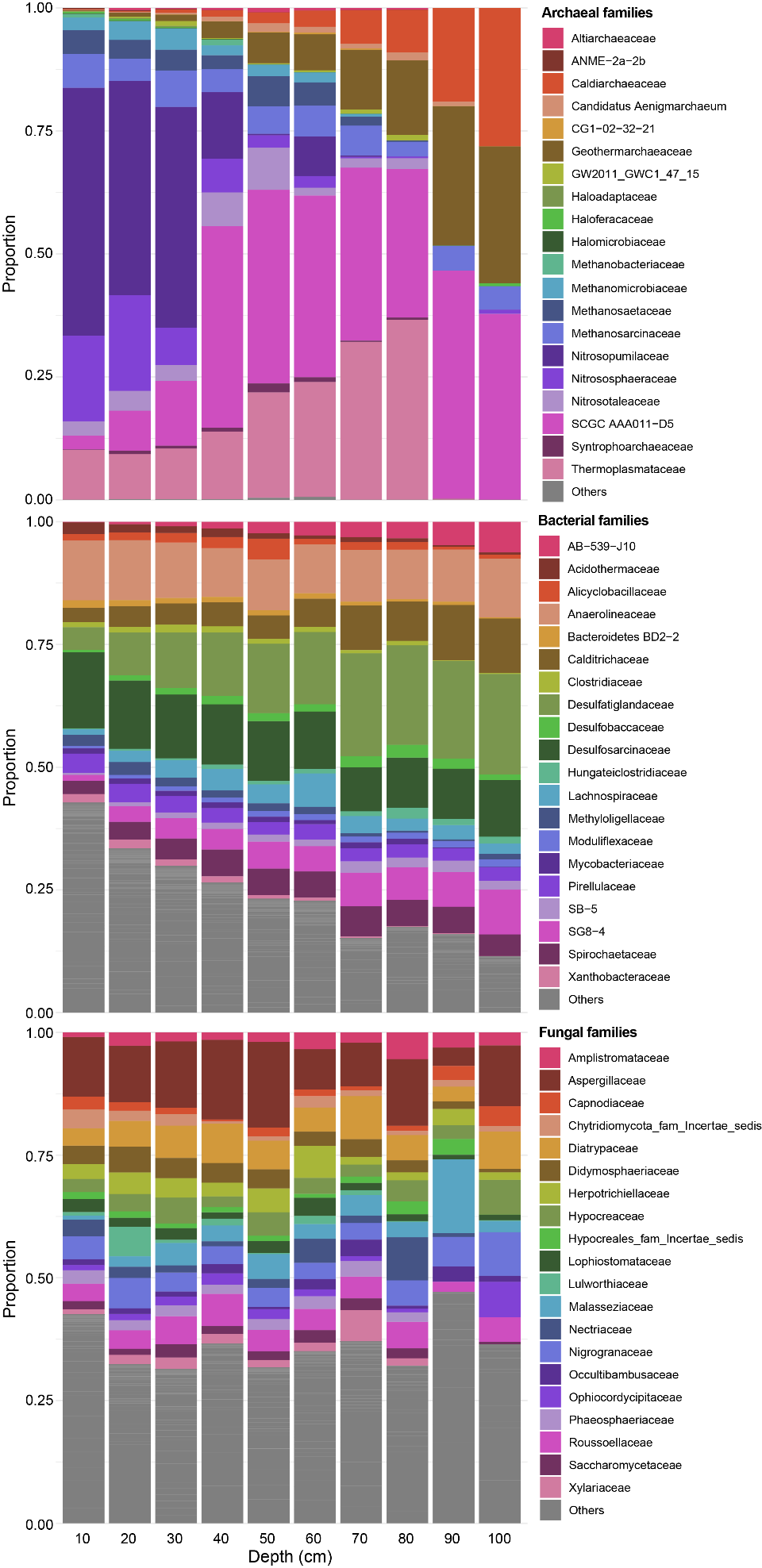


Figure S4. Composition of archaeal, bacterial, and fungal families across sediment depth. Only ASVs that could be identified to family level are represented here.


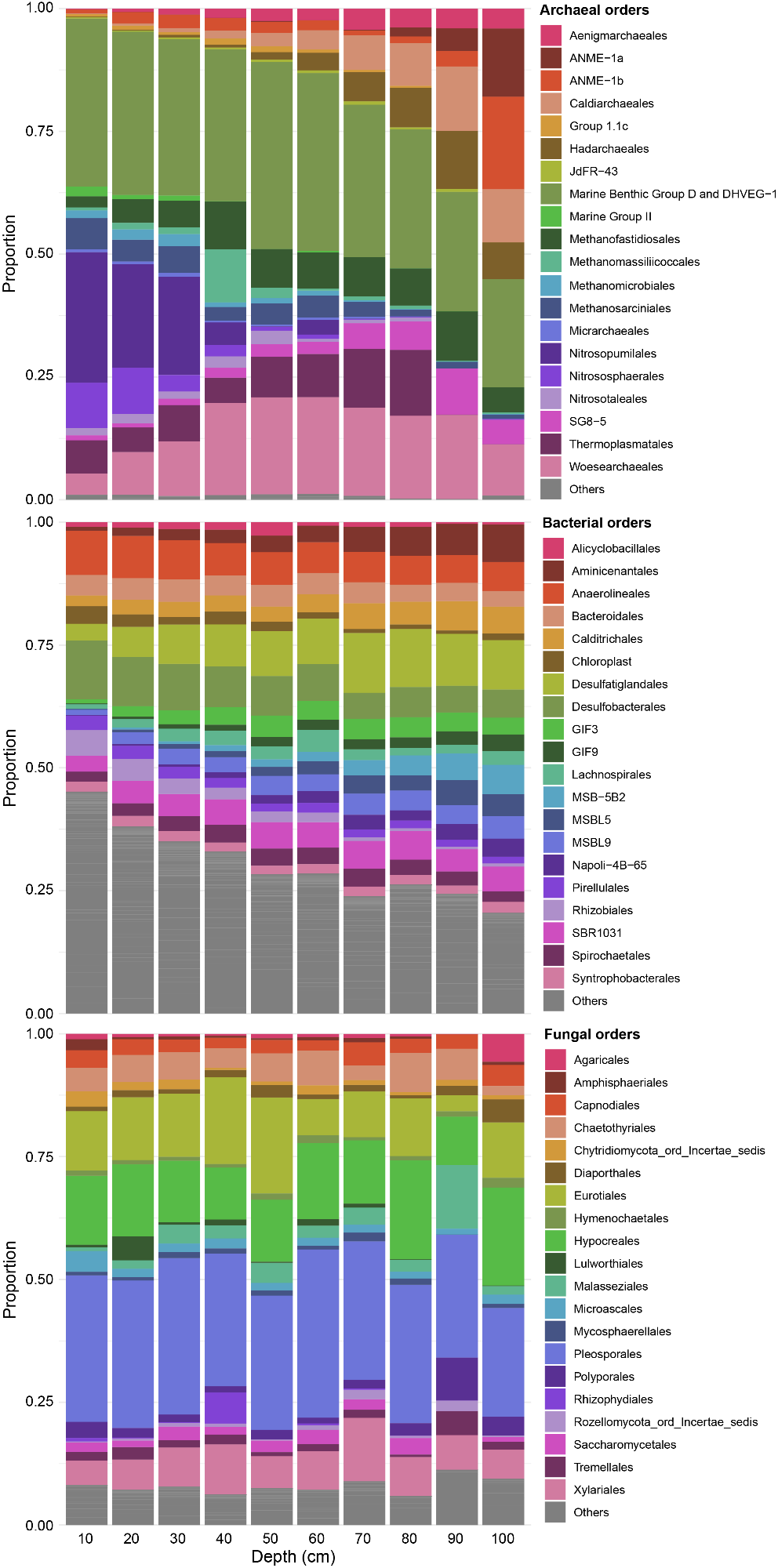


Figure S5. Composition of archaeal, bacterial, and fungal orders across sediment depth. Only ASVs that could be identified to order level are represented here.


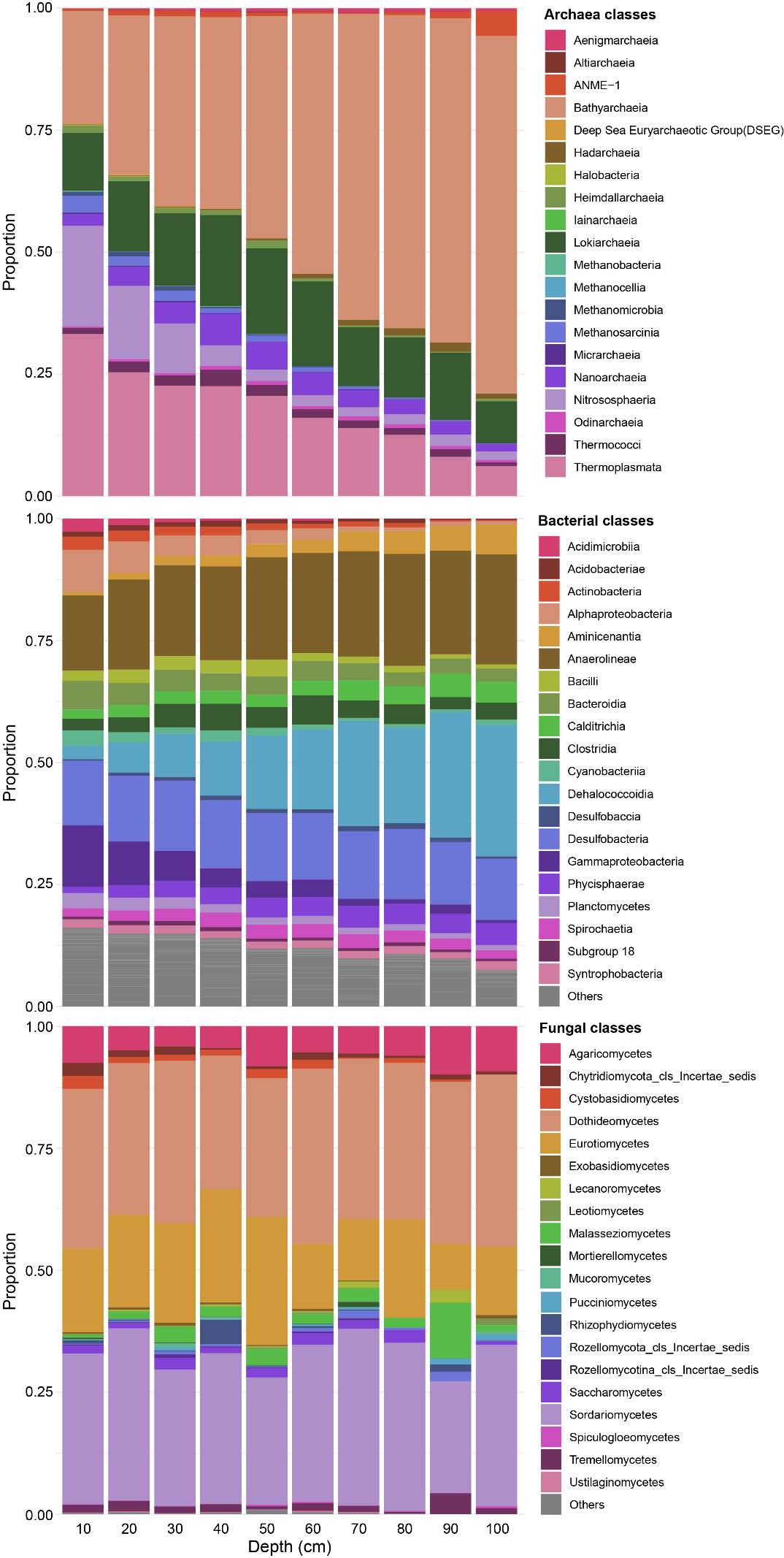


Figure S6. Composition of archaeal, bacterial, and fungal classes across sediment depth. Only ASVs that could be identified to class level are represented here.

Table S1. Number of samples collected at each depth and each site. In all cases, the sediment corer was pushed as far as possible to the ground. However, due to obstruction such as rocks and roots, and the frequent detachment of sediments particularly at the bottom sections, it was not always possible to collect a full 1 m core.

| Depth (cm) | Chek Jawa | Jalan Durian | Ubin Living Lab |
| --- | --- | --- | --- |
| 10 | 10 | 10 | 10 |
| 20 | 10 | 9 | 10 |
| 30 | 10 | 9 | 10 |
| 40 | 9 | 6 | 10 |
| 50 | 7 | 5 | 10 |
| 60 | 6 | 4 | 9 |
| 70 | 5 | 4 | 9 |
| 80 | 3 | 2 | 6 |
| 90 | 2 | 0 | 5 |
| 100 | 0 | 0 | 3 |
| Total | 62 | 49 | 82 |

Table S2. Number of sequencing reads per sample before and after the processing pipeline.

|  | 16S | | | ITS | | |
| --- | --- | --- | --- | --- | --- | --- |
| sampleID | reads.in | reads.out | no.chim | reads.in | reads.out | no.chim |
| CJ1_10 | 142496 | 94460 | 68770 | 59679 | 46550 | 44275 |
| CJ1_20 | 166115 | 105822 | 78939 | 73821 | 56220 | 53471 |
| CJ1_30 | 143057 | 97141 | 75283 | 18326 | 12806 | 12187 |
| CJ1_40 | 217880 | 144795 | 118720 | 31432 | 23917 | 21879 |
| CJ1_50 | 158769 | 99741 | 81136 | 32952 | 21641 | 19231 |
| CJ10_10 | 91127 | 61624 | 28924 | 25117 | 18863 | 17516 |
| CJ10_20 | 82066 | 55346 | 24814 | 30058 | 19636 | 17947 |
| CJ10_30 | 95704 | 62470 | 30485 | 17160 | 11923 | 11426 |
| CJ10_40 | 87373 | 58408 | 26717 | 5707 | 4090 | 3559 |
| CJ10_50 | 88394 | 57780 | 27891 | 19741 | 15050 | 14600 |
| CJ10_60 | 112662 | 71308 | 38561 | 63719 | 46284 | 45234 |
| CJ10_70 | 160471 | 108532 | 58446 | 17213 | 13620 | 12380 |
| CJ10_80 | 85718 | 53961 | 29057 | 1973 | 1321 | 1149 |
| CJ10_90 | 86169 | 55633 | 31143 | 2434 | 1789 | 1366 |
| CJ2_10 | 223074 | 144535 | 112232 | 38973 | 31154 | 28536 |
| CJ2_20 | 132010 | 86336 | 71526 | 31492 | 21796 | 17926 |
| CJ2_30 | 49752 | 29394 | 27285 | 27409 | 17919 | 16461 |
| CJ2_40 | 57867 | 40971 | 26031 | 20208 | 15958 | 14976 |
| CJ3_10 | 6526 | 4518 | 1915 | 72126 | 60724 | 56081 |
| CJ3_20 | 64791 | 39935 | 26965 | 28347 | 21928 | 19942 |
| CJ3_30 | 107917 | 76760 | 57143 | 37727 | 30170 | 27190 |
| CJ4_10 | 141695 | 94833 | 64542 | 35749 | 28633 | 26922 |
| CJ4_20 | 74847 | 46448 | 31272 | 5487 | 4067 | 3339 |
| CJ4_30 | 76109 | 45881 | 42699 | 33575 | 24887 | 24042 |
| CJ4_40 | 97598 | 64519 | 44042 | 27881 | 21387 | 20498 |
| CJ4_50 | 92907 | 60643 | 36786 | 25444 | 18134 | 17071 |
| CJ4_60 | 158666 | 113403 | 71527 | 11967 | 8632 | 5104 |
| CJ5_10 | 99714 | 67439 | 48247 | 33649 | 25988 | 24613 |
| CJ5_20 | 84735 | 52347 | 40007 | 15422 | 11734 | 9855 |
| CJ5_30 | 54605 | 33509 | 26168 | 37299 | 27607 | 25229 |
| CJ5_40 | 123002 | 81328 | 70699 | 27645 | 21307 | 20311 |
| CJ6_10 | 92428 | 64052 | 34520 | 36163 | 26331 | 23656 |
| CJ6_20 | 91532 | 60853 | 33408 | 31298 | 25347 | 22880 |
| CJ6_30 | 94085 | 59455 | 30741 | 20625 | 17331 | 14709 |
| CJ6_40 | 62240 | 41257 | 23443 | 22522 | 16719 | 15591 |
| CJ6_50 | 90466 | 65057 | 33711 | 24999 | 14307 | 13159 |
| CJ6_60 | 45750 | 30316 | 17235 | 25219 | 19855 | 18305 |
| CJ6_70 | 138762 | 97124 | 65529 | 16846 | 5131 | 4527 |
| CJ6_80 | 84291 | 52828 | 28991 | 14698 | 11951 | 10115 |
| CJ7_10 | 115159 | 75306 | 40844 | 28440 | 18181 | 16270 |
| CJ7_20 | 82214 | 54447 | 23465 | 53695 | 44209 | 39859 |
| CJ7_30 | 104392 | 75615 | 36696 | 40371 | 32474 | 29392 |
| CJ7_40 | 128623 | 88551 | 52193 | 27149 | 21480 | 19688 |
| CJ7_50 | 136454 | 87771 | 50013 | 10176 | 8446 | 7581 |
| CJ7_60 | 102701 | 66591 | 34614 | 31279 | 25370 | 23111 |
| CJ7_70 | 102602 | 60138 | 35973 | 37063 | 28760 | 25221 |
| CJ8_10 | 63231 | 44611 | 19421 | 32674 | 26323 | 23800 |
| CJ8_20 | 139970 | 89700 | 60190 | 18895 | 11505 | 10396 |
| CJ8_30 | 102014 | 68363 | 43706 | 36073 | 28694 | 25352 |
| CJ8_40 | 122768 | 82378 | 58722 | 14240 | 11438 | 10584 |
| CJ8_50 | 75965 | 49852 | 26490 | 17494 | 13684 | 12222 |
| CJ8_60 | 82619 | 51856 | 33211 | 15900 | 13005 | 11712 |
| CJ8_70 | 99916 | 68355 | 44474 | 28435 | 21983 | 19867 |
| CJ9_10 | 164783 | 110319 | 67870 | 9755 | 7784 | 7014 |
| CJ9_20 | 76788 | 50882 | 22989 | 35163 | 25652 | 23153 |
| CJ9_30 | 79098 | 50740 | 24266 | 29283 | 22797 | 20171 |
| CJ9_40 | 91968 | 57989 | 29190 | 41257 | 31260 | 29670 |
| CJ9_50 | 85680 | 57463 | 30945 | 23585 | 17638 | 15439 |
| CJ9_60 | 112673 | 72511 | 37100 | 34766 | 27108 | 26059 |
| CJ9_70 | 94887 | 62973 | 33883 | 58035 | 43071 | 41769 |
| CJ9_80 | 83415 | 50199 | 24764 | 40246 | 31935 | 29584 |
| CJ9_90 | 55795 | 35237 | 21064 | 15858 | 11354 | 9640 |
| JD1_10 | 58690 | 38691 | 11874 | 12645 | 10495 | 8573 |
| JD10_10 | 72719 | 49300 | 24992 | 10173 | 6055 | 4685 |
| JD10_20 | 61953 | 43326 | 22650 | 55300 | 39574 | 35578 |
| JD10_30 | 66225 | 45028 | 22339 | 39156 | 31672 | 29157 |
| JD2_10 | 82214 | 50399 | 36651 | 33201 | 25723 | 22937 |
| JD2_20 | 61745 | 41061 | 26865 | 23104 | 15652 | 13978 |
| JD2_30 | 117651 | 77944 | 56218 | 14110 | 11570 | 10771 |
| JD2_40 | 103483 | 68866 | 53808 | 18490 | 15639 | 14863 |
| JD2_50 | 157758 | 98691 | 76112 | 20447 | 17408 | 16897 |
| JD2_60 | 83972 | 52862 | 42452 | 8579 | 6937 | 6723 |
| JD2_70 | 98309 | 63840 | 47344 | 22973 | 14818 | 14495 |
| JD2_80 | 66642 | 43537 | 35470 | 32223 | 24422 | 23335 |
| JD3_10 | 91625 | 59279 | 30692 | 30378 | 24079 | 22005 |
| JD3_20 | 61392 | 43918 | 20547 | 19842 | 15393 | 14466 |
| JD3_30 | 97215 | 65238 | 32951 | 34734 | 28176 | 26721 |
| JD3_40 | 85340 | 56765 | 27474 | 22980 | 17119 | 15732 |
| JD3_50 | 92470 | 61237 | 35644 | 13404 | 10617 | 9954 |
| JD3_60 | 76990 | 51873 | 30601 | 22064 | 17268 | 16475 |
| JD3_70 | 133252 | 86114 | 49915 | 27049 | 16275 | 15158 |
| JD4_10 | 51305 | 33312 | 17475 | 56102 | 40184 | 37657 |
| JD4_20 | 112592 | 81718 | 40215 | 49856 | 37423 | 35777 |
| JD4_30 | 93181 | 66098 | 31718 | 47782 | 32897 | 29979 |
| JD5_10 | 79410 | 49958 | 21122 | 66405 | 50447 | 47401 |
| JD5_20 | 159245 | 105430 | 57010 | 16077 | 12181 | 10886 |
| JD5_30 | 145709 | 94194 | 50684 | 42003 | 31688 | 29653 |
| JD5_40 | 140748 | 94074 | 51413 | 9904 | 6483 | 5936 |
| JD5_50 | 157856 | 98648 | 47575 | 5593 | 3273 | 2964 |
| JD5_60 | 142712 | 98038 | 53563 | 6780 | 4983 | 4671 |
| JD5_70 | 90361 | 58626 | 28166 | 13693 | 10036 | 9606 |
| JD5_80 | 119861 | 83883 | 47055 | 10943 | 7899 | 7670 |
| JD6_10 | 147326 | 96230 | 68909 | 11027 | 8727 | 8104 |
| JD6_20 | 107243 | 76892 | 47434 | 24044 | 19165 | 18117 |
| JD6_30 | 54376 | 37203 | 26208 | 30165 | 22970 | 21705 |
| JD6_40 | 81870 | 52014 | 36411 | 30831 | 22217 | 21358 |
| JD7_10 | 68070 | 46110 | 23055 | 36217 | 28304 | 24908 |
| JD7_20 | 94825 | 64105 | 34225 | 22726 | 16879 | 15684 |
| JD7_30 | 100880 | 68539 | 38359 | 50234 | 34203 | 32640 |
| JD8_10 | 100934 | 73988 | 36231 | 15756 | 10280 | 9091 |
| JD8_20 | 37228 | 26680 | 11610 | 30823 | 22874 | 21335 |
| JD8_30 | 10818 | 7286 | 2288 | 18422 | 11002 | 10489 |
| JD8_40 | 29636 | 19629 | 9404 | 42976 | 30174 | 28773 |
| JD8_50 | 126838 | 88245 | 51613 | 38125 | 21175 | 19584 |
| JD8_60 | 114840 | 74536 | 41925 | 58772 | 48356 | 45615 |
| JD8_70 | 120185 | 77736 | 47456 | 60125 | 47368 | 45658 |
| JD9_10 | 84172 | 57661 | 30492 | 51607 | 39251 | 35204 |
| JD9_20 | 66932 | 46524 | 25031 | 62337 | 49312 | 46444 |
| JD9_30 | 63740 | 42681 | 22940 | 48327 | 38115 | 35838 |
| JD9_40 | 94636 | 62369 | 34136 | 46281 | 35337 | 32908 |
| JD9_50 | 62039 | 41306 | 21060 | 51829 | 39704 | 37396 |
| MOCK | 57280 | 38890 | 31585 | 14474 | 12156 | 10613 |
| UL1_10 | 77603 | 53071 | 34928 | 21449 | 16375 | 15074 |
| UL1_20 | 101555 | 68971 | 39502 | 29623 | 24442 | 22785 |
| UL1_30 | 132599 | 87439 | 59381 | 45459 | 34325 | 32654 |
| UL1_40 | 102185 | 70031 | 43690 | 73663 | 58457 | 56307 |
| UL1_50 | 98467 | 67083 | 45497 | 11596 | 9152 | 8749 |
| UL1_60 | 107038 | 68458 | 47410 | 27547 | 15413 | 13687 |
| UL1_70 | 65764 | 42567 | 23444 | 64847 | 47345 | 43262 |
| UL1_80 | 55870 | 37387 | 19049 | 51738 | 35101 | 32724 |
| UL10_10 | 40880 | 27460 | 13214 | 20398 | 16101 | 14907 |
| UL10_20 | 32227 | 21101 | 10432 | 23647 | 18542 | 17223 |
| UL10_30 | 117476 | 79747 | 38050 | 27154 | 21254 | 19810 |
| UL10_40 | 74793 | 49447 | 23029 | 34560 | 26086 | 24649 |
| UL10_50 | 104104 | 66264 | 32878 | 22699 | 16799 | 16305 |
| UL10_60 | 63710 | 42901 | 21704 | 38321 | 30229 | 27146 |
| UL10_70 | 92261 | 63188 | 36768 | 13964 | 10243 | 9552 |
| UL2_10 | 114263 | 74648 | 42520 | 45016 | 32374 | 30259 |
| UL2_20 | 89507 | 62090 | 39639 | 11671 | 8242 | 7481 |
| UL2_30 | 81061 | 54424 | 43239 | 13976 | 9035 | 8286 |
| UL2_40 | 75983 | 47826 | 31967 | 1801 | 1046 | 654 |
| UL2_50 | 74658 | 50783 | 37171 | 2218 | 1202 | 836 |
| UL2_60 | 67652 | 42273 | 23401 | 5076 | 1635 | 1280 |
| UL2_70 | 78500 | 49621 | 33579 | 12478 | 9166 | 8398 |
| UL3_10 | 65656 | 44031 | 25168 | 23780 | 18154 | 16610 |
| UL3_100 | 69917 | 43158 | 29836 | 21077 | 16142 | 15428 |
| UL3_20 | 61719 | 38704 | 24731 | 14574 | 10815 | 10020 |
| UL3_30 | 104200 | 68273 | 50996 | 32269 | 25076 | 23251 |
| UL3_40 | 117420 | 73722 | 52500 | 9449 | 4371 | 4069 |
| UL3_50 | 65991 | 44467 | 29227 | 13024 | 8193 | 7445 |
| UL3_60 | 85821 | 58453 | 42458 | 44758 | 32706 | 29423 |
| UL3_70 | 64698 | 41220 | 28403 | 35250 | 20352 | 18368 |
| UL3_80 | 60043 | 41722 | 30254 | 23009 | 17878 | 17260 |
| UL3_90 | 71497 | 47611 | 33960 | 27090 | 20876 | 19583 |
| UL4_10 | 58539 | 37112 | 18715 | 37202 | 29245 | 26367 |
| UL4_20 | 26403 | 17028 | 7899 | 30086 | 23667 | 22195 |
| UL4_30 | 52569 | 36859 | 18211 | 29238 | 23987 | 22520 |
| UL4_40 | 42659 | 28182 | 14897 | 38846 | 28275 | 27178 |
| UL4_50 | 115460 | 76977 | 47094 | 18584 | 10863 | 10477 |
| UL4_60 | 69617 | 45910 | 24371 | 14017 | 11046 | 10017 |
| UL4_70 | 68751 | 38821 | 21646 | 18609 | 12175 | 11529 |
| UL4_80 | 63865 | 41343 | 24168 | 12328 | 9005 | 8866 |
| UL4_90 | 69525 | 45381 | 26959 | 27008 | 20959 | 20469 |
| UL5_10 | 46820 | 33476 | 17176 | 1193 | 670 | 539 |
| UL5_20 | 54817 | 37693 | 23571 | 42570 | 31484 | 30315 |
| UL5_30 | 50353 | 34368 | 19898 | 52228 | 39234 | 37792 |
| UL5_40 | 62903 | 41370 | 27610 | 61974 | 32581 | 30248 |
| UL5_50 | 85934 | 57618 | 39543 | 62085 | 42440 | 39254 |
| UL6_10 | 50803 | 33262 | 18735 | 73820 | 54871 | 51146 |
| UL6_100 | 70635 | 46758 | 28649 | 17992 | 14158 | 14048 |
| UL6_20 | 74102 | 50217 | 29286 | 25546 | 18557 | 17542 |
| UL6_30 | 83637 | 54424 | 31981 | 60483 | 45612 | 44117 |
| UL6_40 | 39318 | 25057 | 12425 | 21969 | 16913 | 16481 |
| UL6_50 | 75449 | 49871 | 30811 | 34409 | 23874 | 21486 |
| UL6_60 | 105565 | 68444 | 44171 | 36523 | 27526 | 27146 |
| UL6_70 | 103393 | 65192 | 43667 | 40785 | 24699 | 23710 |
| UL6_80 | 97074 | 62841 | 44095 | 27458 | 20882 | 19552 |
| UL6_90 | 88171 | 54965 | 36242 | 49876 | 37436 | 34361 |
| UL7_10 | 81554 | 51962 | 32299 | 30721 | 24335 | 23318 |
| UL7_100 | 116801 | 71713 | 45290 | 11848 | 9122 | 8865 |
| UL7_20 | 59158 | 38494 | 20285 | 11507 | 9099 | 8770 |
| UL7_30 | 136256 | 94600 | 61169 | 17 | 9 | 1 |
| UL7_40 | 52678 | 35264 | 25272 | 20042 | 16051 | 15469 |
| UL7_50 | 114837 | 71973 | 47284 | 33089 | 20295 | 19872 |
| UL7_60 | 83863 | 52610 | 32379 | 35103 | 27752 | 26457 |
| UL7_70 | 72314 | 46739 | 27309 | 53006 | 41227 | 37606 |
| UL7_80 | 89088 | 57983 | 36982 | 28472 | 21475 | 20547 |
| UL7_90 | 72985 | 43844 | 28423 | 47648 | 37180 | 35521 |
| UL8_10 | 129155 | 91217 | 52648 | 17409 | 13972 | 12955 |
| UL8_20 | 37204 | 25386 | 13106 | 20298 | 16105 | 15625 |
| UL8_30 | 130093 | 84351 | 47902 | 27383 | 15863 | 15436 |
| UL8_40 | 79827 | 55671 | 40736 | 38843 | 29475 | 27921 |
| UL8_50 | 69637 | 45832 | 31550 | 33178 | 22263 | 21814 |
| UL8_60 | 59831 | 40440 | 35786 | 29904 | 21398 | 20962 |
| UL8_70 | 147104 | 99727 | 71249 | 43376 | 32907 | 32155 |
| UL8_80 | 149549 | 98409 | 66492 | 17474 | 13418 | 12800 |
| UL8_90 | 61249 | 40387 | 22020 | 12076 | 8895 | 8743 |
| UL9_10 | 38923 | 25253 | 13404 | 7423 | 5595 | 5242 |
| UL9_20 | 138997 | 94959 | 61634 | 18860 | 5778 | 5366 |
| UL9_30 | 99842 | 65283 | 41538 | 44967 | 33485 | 31988 |
| UL9_40 | 109565 | 72307 | 40776 | 40463 | 23283 | 22596 |
| UL9_50 | 109601 | 70661 | 40652 | 21000 | 16118 | 15539 |
| UL9_60 | 66414 | 43376 | 22330 | 65384 | 51747 | 50925 |
| UL9_70 | 77181 | 50175 | 30857 | 15120 | 11429 | 11167 |

Table S3. Successful taxonomic assignment of each microbial group at each taxonomic level.

|  | Archaea | Bacteria | Fungi |
| --- | --- | --- | --- |
| Phylum | 91.27% | 89.88% | 66.91% |
| Class | 89.04% | 81.07% | 56.82% |
| Order | 66.18% | 64.75% | 50.22% |
| Family | 22.03% | 42.21% | 42.72% |
| Genus | 3.44% | 19.00% | 34.74% |

Table S4. Linear mixed effects models (LME) were conducted to investigate if the alpha diversity indices, Shannon diversity, Richness, and Evenness, of the archaeal, bacterial, and fungal community were significantly changing with sediment depth and across sampling sites. Sediment cores were accounted as the random effect.

|  | Alpha-diversity | Variable | numDF | denDF | F-value | *p-*value |
| --- | --- | --- | --- | --- | --- | --- |
| Archaea | Shannon | Intercept | 1 | 162 | 1252.9997 | <.0001 |
|  |  | Depth | 1 | 162 | 58.8721 | <.0001 |
|  |  | Site | 2 | 27 | 1.4952 | 0.2422 |
|  | Richness | Intercept | 1 | 162 | 262.7471 | <.0001 |
|  |  | Depth | 1 | 162 | 83.4177 | <.0001 |
|  |  | Site | 2 | 27 | 0.1263 | 0.8819 |
|  | Evenness | Intercept | 1 | 162 | 923.6773 | <.0001 |
|  |  | Depth | 1 | 162 | 64.2694 | <.0001 |
|  |  | Site | 2 | 27 | 2.8555 | 0.075 |
| Bacteria | Shannon | Intercept | 1 | 162 | 13158.936 | <.0001 |
|  |  | Depth | 1 | 162 | 1.596 | 0.2083 |
|  |  | Site | 2 | 27 | 0.365 | 0.6973 |
|  | Richness | Intercept | 1 | 162 | 35410.01 | <.0001 |
|  |  | Depth | 1 | 162 | 1.43 | 0.2329 |
|  |  | Site | 2 | 27 | 0.26 | 0.7760 |
|  | Evenness | Intercept | 1 | 162 | 630.3049 | <.0001 |
|  |  | Depth | 1 | 162 | 1.3397 | 0.2488 |
|  |  | Site | 2 | 27 | 1.6355 | 0.2136 |
| Fungi | Shannon | Intercept | 1 | 161 | 938.6455 | <.0001 |
|  |  | Depth | 1 | 161 | 25.6178 | <.0001 |
|  |  | Site | 2 | 27 | 8.2506 | 0.0016 |
|  | Richness | Intercept | 1 | 161 | 117.96535 | <.0001 |
|  |  | Depth | 1 | 161 | 55.68515 | <.0001 |
|  |  | Site | 2 | 27 | 8.66109 | 0.0012 |
|  | Evenness | Intercept | 1 | 161 | 285.11320 | <.0001 |
|  |  | Depth | 1 | 161 | 44.10600 | <.0001 |
|  |  | Site | 2 | 27 | 3.89172 | 0.0327 |

Table S5. Alpha-diversity indices of the fungal communities of the three sampled mangrove sites.

| Site | Shannon Diversity | Richness | Evenness |
| --- | --- | --- | --- |
| Chek Jawa | 3.76 ± 0.11 | 150 ± 13 | 0.255 ± 0.024 |
| Jalan Durian | 4.62 ± 0.17 | 331 ± 34 | 0.264 ± 0.021 |
| Ubin Living Lab | 4.69 ± 0.10 | 230 ± 17 | 0.452 ± 0.021 |

Table S6. Permutational analysis of variance (PERMANOVA) was conducted to investigate if the archaeal, bacterial, and fungal communities were significantly different across sediment depths, sampling sites, and their interaction effects. Permutations were stratified to their sediment core.

| Kingdom | Factors | Df | SumOfSqs | R^2^ | F | *p*-value |
| --- | --- | --- | --- | --- | --- | --- |
| Archaea | Depth | 9 | 1823.3 | 0.10702 | 2.5186 | 0.001 |
|  | Site | 2 | 655.1 | 0.03845 | 4.0723 | 0.344 |
|  | Residuals | 181 | 14558.8 | 0.85453 |  |  |
|  | Total | 192 | 17037.2 | 1.00000 |  |  |
| Bacteria | Depth | 9 | 4044 | 0.07111 | 1.6213 | 0.001 |
|  | Site | 2 | 2666 | 0.04688 | 4.8100 | 0.001 |
|  | Residuals | 181 | 50165 | 0.88202 |  |  |
|  | Total | 192 | 56876 | 1.00000 |  |  |
| Fungi | Depth | 9 | 673.2 | 0.04483 | 1.0074 | 0.015 |
|  | Site | 2 | 866.2 | 0.05768 | 5.7846 | 0.168 |
|  | Residuals | 180 | 13477.4 | 0.89748 |  |  |
|  | Total | 191 | 15016.8 | 1.00000 |  |  |

Table S7. Results of post-hoc pairwise analysis to identify sediment depth pairs with significantly different archaeal communities, with p-values adjusted for false discovery rate.

| Pairs | SumsOfSqs | F-value | R^2^ | *p*-value | Adj *p-*value |
| --- | --- | --- | --- | --- | --- |
| 10 vs 20 | 85.728 | 1.260 | 0.0216 | 0.064 | 0.08727 |
| 10 vs 30 | 87.699 | 0.853 | 0.0161 | 0.864 | 0.92571 |
| 10 vs 40 | 193.863 | 2.457 | 0.0443 | 0.001 | 0.00225 |
| 10 vs 50 | 308.143 | 3.611 | 0.0674 | 0.001 | 0.00225 |
| 10 vs 60 | 329.499 | 4.123 | 0.0807 | 0.001 | 0.00225 |
| 10 vs 70 | 618.718 | 7.272 | 0.1365 | 0.001 | 0.00225 |
| 10 vs 80 | 439.462 | 5.977 | 0.1329 | 0.001 | 0.00225 |
| 10 vs 90 | 347.398 | 5.388 | 0.1334 | 0.001 | 0.00225 |
| 10 vs 100 | 276.694 | 4.465 | 0.1259 | 0.001 | 0.00225 |
| 20 vs 30 | 85.708 | 0.952 | 0.0167 | 0.551 | 0.65250 |
| 20 vs 40 | 115.019 | 1.269 | 0.0238 | 0.037 | 0.04469 |
| 20 vs 50 | 193.156 | 1.972 | 0.0387 | 0.001 | 0.00225 |
| 20 vs 60 | 232.013 | 2.488 | 0.0513 | 0.001 | 0.00225 |
| 20 vs 70 | 493.908 | 4.998 | 0.1000 | 0.001 | 0.00225 |
| 20 vs 80 | 365.389 | 4.083 | 0.0970 | 0.001 | 0.00225 |
| 20 vs 90 | 310.573 | 3.785 | 0.1002 | 0.001 | 0.00225 |
| 20 vs 100 | 254.412 | 3.110 | 0.0939 | 0.001 | 0.00225 |
| 30 vs 40 | 167.006 | 2.111 | 0.0357 | 0.001 | 0.00225 |
| 30 vs 50 | 165.870 | 1.8334 | 0.0335 | 0.001 | 0.00225 |
| 30 vs 60 | 164.530 | 1.538 | 0.0324 | 0.003 | 0.00614 |
| 30 vs 70 | 392.938 | 3.482 | 0.0718 | 0.001 | 0.00225 |
| 30 vs 80 | 300.782 | 2.835 | 0.0694 | 0.001 | 0.00225 |
| 30 vs 90 | 281.804 | 2.801 | 0.0761 | 0.001 | 0.00225 |
| 30 vs 100 | 234.009 | 2.275 | 0.0705 | 0.001 | 0.00225 |
| 40 vs 50 | 100.148 | 0.883 | 0.0193 | 0.793 | 0.87037 |
| 40 vs 60 | 133.972 | 1.226 | 0.0284 | 0.069 | 0.09132 |
| 40 vs 70 | 320.498 | 2.767 | 0.0632 | 0.001 | 0.00225 |
| 40 vs 80 | 249.464 | 2.290 | 0.0631 | 0.001 | 0.00225 |
| 40 vs 90 | 234.343 | 2.274 | 0.0704 | 0.002 | 0.00429 |
| 40 vs 100 | 212.548 | 2.005 | 0.0716 | 0.013 | 0.02017 |
| 50 vs 60 | 102.317 | 0.853 | 0.0214 | 0.889 | 0.93035 |
| 50 vs 70 | 241.310 | 1.897 | 0.0475 | 0.004 | 0.00783 |
| 50 vs 80 | 199.585 | 1.633 | 0.0500 | 0.006 | 0.01038 |
| 50 vs 90 | 211.613 | 1.798 | 0.0624 | 0.005 | 0.00900 |
| 50 vs 100 | 200.103 | 1.620 | 0.0658 | 0.008 | 0.01333 |
| 60 vs 70 | 175.528 | 1.421 | 0.0390 | 0.01 | 0.01607 |
| 60 vs 80 | 153.148 | 1.308 | 0.0446 | 0.044 | 0.06387 |
| 60 vs 90 | 167.719 | 1.510 | 0.0592 | 0.005 | 0.00900 |
| 60 vs 100 | 171.099 | 1.468 | 0.0684 | 0.022 | 0.03300 |
| 70 vs 80 | 97.255 | 0.764 | 0.0275 | 0.993 | 0.99300 |
| 70 vs 90 | 123.481 | 1.006 | 0.0419 | 0.426 | 0.51811 |
| 70 vs 100 | 134.179 | 1.024 | 0.0511 | 0.41 | 0.51250 |
| 80 vs 90 | 91.683 | 0.825 | 0.0490 | 0.932 | 0.95318 |
| 80 vs 100 | 113.699 | 0.946 | 0.0730 | 0.593 | 0.68423 |
| 90 vs 100 | 95.530 | 0.919 | 0.1030 | 0.661 | 0.74363 |

Table S8. Results of post-hoc pairwise analysis to identify sediment depth pairs with significantly different bacterial communities, with p-values adjusted for false discovery rate.

| Pairs | SumsOfSqs | F-value | R^2^ | *p*-value | Adj *p-*value |
| --- | --- | --- | --- | --- | --- |
| 10 vs 20 | 402.506 | 1.116 | 0.0192 | 0.174 | 0.27964 |
| 10 vs 30 | 288.013 | 0.804 | 0.0152 | 0.987 | 0.99600 |
| 10 vs 40 | 742.055 | 2.037 | 0.0370 | 0.001 | 0.00409 |
| 10 vs 50 | 857.765 | 2.326 | 0.0445 | 0.001 | 0.00409 |
| 10 vs 60 | 760.096 | 2.121 | 0.0432 | 0.001 | 0.00409 |
| 10 vs 70 | 1256.788 | 3.646 | 0.0734 | 0.001 | 0.00409 |
| 10 vs 80 | 913.936 | 2.636 | 0.0633 | 0.001 | 0.00409 |
| 10 vs 90 | 667.716 | 1.929 | 0.0522 | 0.004 | 0.01200 |
| 10 vs 100 | 481.302 | 1.317 | 0.0407 | 0.141 | 0.25269 |
| 20 vs 30 | 289.752 | 0.816 | 0.0144 | 0.963 | 0.99600 |
| 20 vs 40 | 418.182 | 1.198 | 0.0225 | 0.037 | 0.07929 |
| 20 vs 50 | 516.568 | 1.465 | 0.0290 | 0.002 | 0.00643 |
| 20 vs 60 | 518.916 | 1.521 | 0.0320 | 0.001 | 0.00409 |
| 20 vs 70 | 945.659 | 2.894 | 0.0604 | 0.001 | 0.00409 |
| 20 vs 80 | 705.539 | 2.168 | 0.0540 | 0.001 | 0.00409 |
| 20 vs 90 | 564.471 | 1.750 | 0.0490 | 0.001 | 0.00409 |
| 20 vs 100 | 420.896 | 1.240 | 0.0397 | 0.165 | 0.27500 |
| 30 vs 40 | 567.316 | 1.537 | 0.0263 | 0.002 | 0.00643 |
| 30 vs 50 | 350.471 | 0.967 | 0.0194 | 0.558 | 0.76091 |
| 30 vs 60 | 380.494 | 1.083 | 0.0230 | 0.199 | 0.30879 |
| 30 vs 70 | 717.696 | 2.128 | 0.0452 | 0.001 | 0.00409 |
| 30 vs 80 | 548.859 | 1.624 | 0.0410 | 0.002 | 0.00643 |
| 30 vs 90 | 489.054 | 1.454 | 0.0410 | 0.007 | 0.01969 |
| 30 vs 100 | 376.241 | 1.060 | 0.0341 | 0.366 | 0.54900 |
| 40 vs 50 | 284.221 | 0.798 | 0.0174 | 0.98 | 0.99600 |
| 40 vs 60 | 330.894 | 0.962 | 0.0224 | 0.588 | 0.77143 |
| 40 vs 70 | 577.280 | 1.759 | 0.0411 | 0.001 | 0.00409 |
| 40 vs 80 | 451.565 | 1.381 | 0.0390 | 0.011 | 0.02912 |
| 40 vs 90 | 417.479 | 1.289 | 0.0412 | 0.028 | 0.06632 |
| 40 vs 100 | 344.560 | 1.003 | 0.0372 | 0.448 | 0.65032 |
| 50 vs 60 | 276.832 | 0.796 | 0.0200 | 0.996 | 0.99600 |
| 50 vs 70 | 463.459 | 1.400 | 0.0355 | 0.012 | 0.03000 |
| 50 vs 80 | 398.811 | 1.207 | 0.0375 | 0.058 | 0.11864 |
| 50 vs 90 | 400.544 | 1.224 | 0.0434 | 0.065 | 0.12717 |
| 50 vs 100 | 325.849 | 0.931 | 0.0389 | 0.600 | 0.77143 |
| 60 vs 70 | 390.594 | 1.244 | 0.0343 | 0.033 | 0.07425 |
| 60 vs 80 | 352.220 | 1.140 | 0.0391 | 0.106 | 0.19875 |
| 60 vs 90 | 345.777 | 1.145 | 0.0455 | 0.146 | 0.25269 |
| 60 vs 100 | 300.097 | 0.929 | 0.0444 | 0.541 | 0.76078 |
| 70 vs 80 | 218.009 | 0.768 | 0.0277 | 0.996 | 0.99600 |
| 70 vs 90 | 234.775 | 0.863 | 0.0362 | 0.862 | 0.99600 |
| 70 vs 100 | 225.283 | 0.782 | 0.0395 | 0.873 | 0.99600 |
| 80 vs 90 | 193.335 | 0.788 | 0.0470 | 0.983 | 0.99600 |
| 80 vs 100 | 194.086 | 0.742 | 0.0582 | 0.972 | 0.99600 |
| 90 vs 100 | 167.050 | 0.770 | 0.0878 | 0.983 | 0.99600 |

Table S9. Results of post-hoc pairwise analysis to identify sediment depth pairs with significantly different fungal communities, with p-values adjusted for false discovery rate.

| Pairs | SumsOfSqs | F-value | R^2^ | *p*-value | Adj *p-*value |
| --- | --- | --- | --- | --- | --- |
| 10 vs 20 | 122.4503 | 0.9230 | 0.0159 | 0.675 | 0.99800 |
| 10 vs 30 | 116.9493 | 0.9177 | 0.0161 | 0.714 | 0.99800 |
| 10 vs 40 | 153.8091 | 1.384 | 0.0255 | 0.012 | 0.54000 |
| 10 vs 50 | 139.0723 | 1.2378 | 0.0242 | 0.058 | 0.59400 |
| 10 vs 60 | 146.8815 | 1.2255 | 0.0254 | 0.066 | 0.59400 |
| 10 vs 70 | 142.8437 | 1.2436 | 0.0263 | 0.060 | 0.59400 |
| 10 vs 80 | 122.5218 | 1.0033 | 0.0251 | 0.421 | 0.99800 |
| 10 vs 90 | 101.6512 | 0.8116 | 0.0227 | 0.812 | 0.99800 |
| 10 vs 100 | 65.9420 | 0.4677 | 0.0149 | 0.988 | 0.99800 |
| 20 vs 30 | 88.4839 | 0.7978 | 0.0143 | 0.988 | 0.99800 |
| 20 vs 40 | 106.7179 | 1.1439 | 0.0215 | 0.161 | 0.72450 |
| 20 vs 50 | 98.7865 | 1.0566 | 0.0211 | 0.254 | 0.87923 |
| 20 vs 60 | 107.7267 | 1.0781 | 0.0229 | 0.249 | 0.87923 |
| 20 vs 70 | 103.9102 | 1.1010 | 0.0239 | 0.221 | 0.87923 |
| 20 vs 80 | 92.6230 | 0.9447 | 0.0243 | 0.545 | 0.99800 |
| 20 vs 90 | 79.8135 | 0.8108 | 0.0233 | 0.731 | 0.99800 |
| 20 vs 100 | 55.6601 | 0.5008 | 0.0164 | 0.970 | 0.99800 |
| 30 vs 40 | 98.1700 | 1.1313 | 0.0217 | 0.143 | 0.72450 |
| 30 vs 50 | 97.9795 | 1.1318 | 0.0230 | 0.152 | 0.72450 |
| 30 vs 60 | 110.297 | 1.1901 | 0.0258 | 0.107 | 0.72450 |
| 30 vs 70 | 100.417 | 1.1563 | 0.0256 | 0.122 | 0.72450 |
| 30 vs 80 | 92.8250 | 1.0407 | 0.0274 | 0.337 | 0.99800 |
| 30 vs 90 | 81.5618 | 0.9214 | 0.0272 | 0.570 | 0.99800 |
| 30 vs 100 | 52.8555 | 0.5270 | 0.0178 | 0.990 | 0.99800 |
| 40 vs 50 | 51.1889 | 0.7923 | 0.0173 | 0.977 | 0.99800 |
| 40 vs 60 | 69.5128 | 0.9990 | 0.0232 | 0.382 | 0.99800 |
| 40 vs 70 | 51.5136 | 0.8209 | 0.0196 | 0.945 | 0.99800 |
| 40 vs 80 | 49.9422 | 0.8276 | 0.0238 | 0.828 | 0.99800 |
| 40 vs 90 | 43.0768 | 0.7725 | 0.0251 | 0.851 | 0.99800 |
| 40 vs 100 | 38.6177 | 0.6048 | 0.0227 | 0.890 | 0.99800 |
| 50 vs 60 | 61.7157 | 0.9075 | 0.0227 | 0.658 | 0.99800 |
| 50 vs 70 | 50.0504 | 0.8259 | 0.0213 | 0.919 | 0.99800 |
| 50 vs 80 | 48.3519 | 0.8413 | 0.0264 | 0.804 | 0.99800 |
| 50 vs 90 | 39.2456 | 0.7554 | 0.0272 | 0.833 | 0.99800 |
| 50 vs 100 | 37.0916 | 0.6137 | 0.0260 | 0.834 | 0.99800 |
| 60 vs 70 | 50.6030 | 0.7641 | 0.0214 | 0.998 | 0.99800 |
| 60 vs 80 | 58.4267 | 0.9105 | 0.0315 | 0.648 | 0.99800 |
| 60 vs 90 | 39.2276 | 0.6639 | 0.0269 | 0.952 | 0.99800 |
| 60 vs 100 | 38.6887 | 0.5506 | 0.0268 | 0.845 | 0.99800 |
| 70 vs 80 | 43.1307 | 0.8046 | 0.0289 | 0.889 | 0.99800 |
| 70 vs 90 | 33.1687 | 0.7140 | 0.0301 | 0.954 | 0.99800 |
| 70 vs 100 | 35.8354 | 0.6449 | 0.0328 | 0.834 | 0.99800 |
| 80 vs 90 | 30.1242 | 0.8806 | 0.0522 | 0.725 | 0.99800 |
| 80 vs 100 | 32.8730 | 0.7379 | 0.0579 | 0.626 | 0.99800 |
| 90 vs 100 | 28.1520 | 1.4464 | 0.1531 | 0.061 | 0.59400 |

Table S10. Linear mixed effects models (LME) were conducted to investigate if the sediment properties (pH, mean particle size, nitrogen %, sulfur %, carbon %, and phosphorus %) were significantly changing with sediment depth and across sampling sites. Sediment cores were accounted as the random effect.

|  |  | numDF | denDF | F-value | t-value | *p*-value |
| --- | --- | --- | --- | --- | --- | --- |
| pH | Intercept | 1 | 162 | 1535.9779 | 23.944551 | <.0001 |
|  | Depth | 1 | 162 | 75.0179 | -8.820912 | <.0001 |
|  | Site | 2 | 27 | 2.1528 |  | 0.1357 |
| Mean_PS | Intercept | 1 | 159 | 329.1956 | 16.679704 | <.0001 |
|  | Depth | 1 | 159 | 34.4650 | -5.362656 | <.0001 |
|  | Site | 2 | 27 | 24.1467 |  | <.0001 |
| Nitrogen | Intercept | 1 | 161 | 244.30403 | 4.197931 | <.0001 |
|  | Depth | 1 | 161 | 1.49012 | -1.378171 | 0.224 |
|  | Site | 2 | 27 | 19.83430 |  | <.0001 |
| Sulfur | Intercept | 1 | 162 | 92.72990 | 0.832225 | <.0001 |
|  | Depth | 1 | 162 | 36.31691 | 5.803533 | <.0001 |
|  | Site | 2 | 27 | 4.88304 |  | 0.0155 |
| Carbon | Intercept | 1 | 162 | 142.21636 | 2.243933 | <.0001 |
|  | Depth | 1 | 162 | 9.14043 | 2.919550 | 0.0029 |
|  | Site | 2 | 27 | 9.70006 |  | 0.0007 |
| Phosphorus | Intercept | 1 | 162 | 474.4450 | 5.135921 | <.0001 |
|  | Depth | 1 | 162 | 36.9387 | -5.775460 | <.0001 |
|  | Site | 2 | 27 | 76.7402 |  | <.0001 |

Table S11. Distance-based redundancy analysis (db-RDA) was conducted to investigate if the archaeal communities were significantly correlated with the five measured environmental variables.

|  | Variable | Df | Variance | F | *p-*value |
| --- | --- | --- | --- | --- | --- |
| Overall | Model | 6 | 9.972 | 3.8574 | 0.001 |
|  | Residual | 184 | 79.660 |  |  |
| Axes | RDA1 | 1 | 5.124 | 12.2947 | 0.001 |
|  | RDA2 | 1 | 1.672 | 3.8901 | 0.001 |
|  | RDA3 | 1 | 1.007 | 2.6757 | 0.001 |
|  | RDA4 | 1 | 0.810 | 1.9271 | 0.003 |
|  | RDA5 | 1 | 0.544 | 1.2863 | 0.235 |
|  | RDA6 | 1 | 0.461 | 1.0707 | 0.344 |
|  | Residual | 184 | 79.660 |  |  |
| Terms | pH | 1 | 3.260 | 7.5289 | 0.001 |
|  | P | 1 | 1.891 | 4.3669 | 0.001 |
|  | N | 1 | 1.465 | 3.3836 | 0.001 |
|  | S | 1 | 1.103 | 2.5478 | 0.001 |
|  | C | 1 | 0.809 | 1.8780 | 0.004 |
|  | Particle size | 1 | 1.439 | 3.3230 | 0.001 |
|  | Residual | 184 | 79.660 |  |  |
|  |  | dbRDA1 | dbRDA2 | dbRDA3 | dbRDA4 |
| Importance of components | pH | -0.7406787 | -0.2416408 | -0.5672758 | 0.21761100 |
|  | P | -0.3347614 | 0.9299045 | -0.1237467 | -0.05402402 |
|  | N | 0.1150185 | 0.8073608 | -0.1508398 | 0.28164762 |
|  | S | 0.6925940 | 0.4753527 | -0.1489666 | 0.47389872 |
|  | C | 0.4072254 | 0.6777356 | 0.1149481 | -0.45481535 |
|  | Particle size | -0.4015255 | -0.4099668 | 0.6873640 | 0.40891754 |

Table S12. Distance-based redundancy analysis (db-RDA) was conducted to investigate if the bacterial communities were significantly correlated with the five measured environmental variables.

|  | Variable | Df | Variance | F | *p-*value |
| --- | --- | --- | --- | --- | --- |
| Overall | Model | 6 | 28.617 | 3.2466 | 0.001 |
|  | Residual | 183 | 268.841 |  |  |
| Axes | RDA1 | 1 | 9.223 | 6.2779 | 0.001 |
|  | RDA2 | 1 | 7.306 | 4.9734 | 0.001 |
|  | RDA3 | 1 | 5.352 | 3.6432 | 0.001 |
|  | RDA4 | 1 | 3.413 | 2.3229 | 0.001 |
|  | RDA5 | 1 | 2.012 | 1.3697 | 0.098 |
|  | RDA6 | 1 | 1.311 | 0.8924 | 0.680 |
|  | Residual | 183 | 268.841 |  |  |
| Terms | pH | 1 | 6.455 | 4.3943 | 0.001 |
|  | P | 1 | 7.293 | 4.9641 | 0.001 |
|  | N | 1 | 4.374 | 2.9774 | 0.001 |
|  | S | 1 | 2.614 | 1.7797 | 0.005 |
|  | C | 1 | 2.103 | 1.4316 | 0.016 |
|  | Particle size | 1 | 5.777 | 3.9324 | 0.001 |
|  | Residual | 183 | 268.841 |  |  |
|  |  | dbRDA1 | dbRDA2 | dbRDA3 | dbRDA4 |
| Importance of components | pH | -0.66257 | -0.00809 | 0.54527 | -0.47016 |
|  | P | -0.32551 | 0.93126 | -0.13974 | 0.05917 |
|  | N | 0.10420 | 0.82053 | -0.27247 | -0.43442 |
|  | S | 0.64296 | 0.38181 | -0.25212 | -0.25602 |
|  | C | 0.32993 | 0.68039 | -0.44562 | -0.43575 |
|  | Particle size | -0.52172 | -0.66057 | -0.49316 | -0.02574 |

Table S13. Distance-based redundancy analysis (db-RDA) was conducted to investigate if the fungal communities were significantly correlated with the five measured environmental variables.

|  | Variable | Df | Variance | F | *p-*value |
| --- | --- | --- | --- | --- | --- |
| Overall | Model | 6 | 7.102 | 2.9938 | 0.001 |
|  | Residual | 183 | 71.959 |  |  |
| Axes | RDA1 | 1 | 4.101 | 10.3725 | 0.001 |
|  | RDA2 | 1 | 1.064 | 2.6913 | 0.013 |
|  | RDA3 | 1 | 0.754 | 1.9060 | 0.080 |
|  | RDA4 | 1 | 0.504 | 1.2744 | 0.471 |
|  | RDA5 | 1 | 0.348 | 0.8810 | 0.925 |
|  | RDA6 | 1 | 0.331 | 0.8375 | 0.635 |
|  | Residual | 183 | 82.247 |  |  |
| Terms | pH | 1 | 0.881 | 2.2294 | 0.001 |
|  | P | 1 | 3.759 | 9.5068 | 0.001 |
|  | N | 1 | 0.919 | 2.3256 | 0.001 |
|  | S | 1 | 0.417 | 1.0553 | 0.386 |
|  | C | 1 | 0.475 | 1.2001 | 0.231 |
|  | Particle size | 1 | 0.651 | 1.6455 | 0.007 |
|  | Residual | 183 | 82.247 |  |  |
|  |  | dbRDA1 | dbRDA2 |  |  |
| Importance of components | pH | 0.2663707 | -0.4760482 |  |  |
|  | P | 0.9696587 | 0.1251840 |  |  |
|  | N | 0.7292043 | -0.3870327 |  |  |
|  | S | 0.1637147 | -0.1206208 |  |  |
|  | C | 0.5787807 | 0.2233753 |  |  |
|  | Particle size | -0.2762052 | -0.3751210 |  |  |

Table S14. The topological features of each constructed co-occurrence network.

|  | Surface | Subsurface | Deep |
| --- | --- | --- | --- |
| Nodes | 1771 | 1595 | 1934 |
| Edges | 34,853 | 28,393 | 43,076 |
| % positive edges | 76.25 | 75.63 | 68.91 |
| Average degree | 39.36 | 35.60 | 26.61 |
| Density | 0.022 | 0.022 | 0.015 |
| Clustering coefficient | 0.067 | 0.067 | 0.057 |

Table S15. Percentage of interactions for each constructed co-occurrence network.

| Network | Interaction | Percentage (%) |
| --- | --- | --- |
| Surface | Archaea-Archaea | 0.9009 |
|  | Bacteria-Archaea | 7.6579 |
|  | Bacteria-Bacteria | 27.6504 |
|  | Fungi-Archaea | 4.3267 |
|  | Fungi-Bacteria | 30.0864 |
|  | Fungi-Fungi | 29.3777 |
| Subsurface | Archaea-Archaea | 0.6621 |
|  | Bacteria-Archaea | 9.5552 |
|  | Bacteria-Bacteria | 35.6637 |
|  | Fungi-Archaea | 4.4025 |
|  | Fungi-Bacteria | 31.2929 |
|  | Fungi-Fungi | 18.4236 |
| Deep | Archaea-Archaea | 0.4629 |
|  | Bacteria-Archaea | 24.1619 |
|  | Bacteria-Bacteria | 34.2952 |
|  | Fungi-Archaea | 6.4212 |
|  | Fungi-Bacteria | 20.6519 |
|  | Fungi-Fungi | 9.8407 |

Table S16. Analysis of variance (ANOVA) was conducted on each of the four network centrality measures to investigate if they were significantly different across kingdom, layer, and the interaction effect.

|  |  | Df | Sum Sq | Mean Sq | F value | Pr(>F) |
| --- | --- | --- | --- | --- | --- | --- |
| Degree | Kingdom | 2 | 3255.208 | 1627.604 | 11.12538 | 1.51E-05 |
|  | Layer | 2 | 70871.3 | 35435.65 | 242.218 | 2.22E-101 |
|  | Kingdom:Layer | 4 | 94440.48 | 23610.12 | 161.3854 | 1.57E-130 |
|  | Residuals | 5291 | 774054.8 | 146.2965 |  |  |
| Eigenvector | Kingdom | 2 | 2.919183 | 1.459591 | 49.11861 | 7.31E-22 |
|  | Layer | 2 | 2.806731 | 1.403365 | 47.22648 | 4.69E-21 |
|  | Kingdom:Layer | 4 | 24.29891 | 6.074729 | 204.4286 | 2.75E-163 |
|  | Residuals | 5291 | 157.2255 | 0.029716 |  |  |
| Closeness | Kingdom | 2 | 0.003464 | 0.001732 | 2.447866 | 0.086576 |
|  | Layer | 2 | 0.057515 | 0.028757 | 40.64363 | 3.04E-18 |
|  | Kingdom:Layer | 4 | 0.593733 | 0.148433 | 209.785 | 2.67E-167 |
|  | Residuals | 5291 | 3.74364 | 0.000708 |  |  |
| Betweenness | Kingdom | 2 | 2.44E+08 | 1.22E+08 | 233.6428 | 5.82E-98 |
|  | Layer | 2 | 1.06E+08 | 53053226 | 101.4961 | 5.57E-44 |
|  | Kingdom:Layer | 4 | 1.07E+08 | 26744612 | 51.1651 | 2.48E-42 |
|  | Residuals | 5289 | 2.76E+09 | 522712 |  |  |

Table S17. Post-hoc pairwise analysis was conducted with Least-Squares Means to identify kingdom pairs with significantly different network centrality metric within each layer.

|  | Layer | Contrast | Estimate | SE | Df | *t-*ratio | *p-*value |
| --- | --- | --- | --- | --- | --- | --- | --- |
| Degree | Surface | Archaea - Bacteria | 1.884325 | 1.128600 | 5291 | 1.669613 | 0.217031 |
|  |  | Archaea - Fungi | -9.18502 | 1.150392 | 5291 | -7.98425 | 1.24E-12 |
|  |  | Bacteria - Fungi | -11.0693 | 0.602996 | 5291 | -18.3572 | 1.20E-12 |
|  | Subsurface | Archaea - Bacteria | -1.40616 | 0.856317 | 5291 | -1.6421 | 0.228081 |
|  |  | Archaea - Fungi | -4.80949 | 0.965362 | 5291 | -4.98206 | 1.94E-06 |
|  |  | Bacteria - Fungi | -3.40334 | 0.716422 | 5291 | -4.75046 | 6.22E-06 |
|  | Deep | Archaea - Bacteria | -0.94214 | 0.735864 | 5291 | -1.28031 | 0.406359 |
|  |  | Archaea - Fungi | 9.672703 | 0.817344 | 5291 | 11.83432 | 1.20E-12 |
|  |  | Bacteria - Fungi | 10.61484 | 0.642098 | 5291 | 16.53148 | 1.20E-12 |
| Eigenvector | Surface | Archaea - Bacteria | 0.031681 | 0.016085 | 5291 | 1.969618 | 0.119875 |
|  |  | Archaea - Fungi | -0.17327 | 0.016395 | 5291 | -10.5684 | 1.22E-12 |
|  |  | Bacteria - Fungi | -0.20495 | 0.008594 | 5291 | -23.8488 | 1.20E-12 |
|  | Subsurface | Archaea - Bacteria | -0.03403 | 0.012204 | 5291 | -2.78859 | 0.01469 |
|  |  | Archaea - Fungi | -0.11207 | 0.013758 | 5291 | -8.1458 | 1.23E-12 |
|  |  | Bacteria - Fungi | -0.07804 | 0.010210 | 5291 | -7.64316 | 1.29E-12 |
|  | Deep | Archaea - Bacteria | -0.00797 | 0.010488 | 5291 | -0.76034 | 0.727364 |
|  |  | Archaea - Fungi | 0.132715 | 0.011649 | 5291 | 11.39302 | 1.20E-12 |
|  |  | Bacteria - Fungi | 0.140689 | 0.009151 | 5291 | 15.37384 | 1.20E-12 |
| Closeness | Surface | Archaea - Bacteria | 0.004452 | 0.002482 | 5291 | 1.793809 | 0.171721 |
|  |  | Archaea - Fungi | -0.01948 | 0.002530 | 5291 | -7.70181 | 1.26E-12 |
|  |  | Bacteria - Fungi | -0.02394 | 0.001326 | 5291 | -18.0509 | 1.20E-12 |
|  | Subsurface | Archaea - Bacteria | -0.00461 | 0.001883 | 5291 | -2.44784 | 0.038277 |
|  |  | Archaea - Fungi | -0.01232 | 0.002123 | 5291 | -5.80122 | 2.09E-08 |
|  |  | Bacteria - Fungi | -0.00771 | 0.001576 | 5291 | -4.89119 | 3.08E-06 |
|  | Deep | Archaea - Bacteria | -0.00089 | 0.001618 | 5291 | -0.54844 | 0.847283 |
|  |  | Archaea - Fungi | 0.028582 | 0.001797 | 5291 | 15.90097 | 1.20E-12 |
|  |  | Bacteria - Fungi | 0.029469 | 0.001412 | 5291 | 20.86928 | 1.20E-12 |
| Betweenness | Surface | Archaea - Bacteria | -19.4348 | 67.56116 | 5291 | -0.28766 | 0.955411 |
|  |  | Archaea - Fungi | -254.46 | 68.86567 | 5291 | -3.69502 | 0.00065 |
|  |  | Bacteria - Fungi | -235.025 | 36.09702 | 5291 | -6.51092 | 2.46E-10 |
|  | Subsurface | Archaea - Bacteria | 14.6362 | 51.26153 | 5291 | 0.28552 | 0.956058 |
|  |  | Archaea - Fungi | -250.098 | 57.78926 | 5291 | -4.32776 | 4.56E-05 |
|  |  | Bacteria - Fungi | -264.734 | 42.88704 | 5291 | -6.17283 | 2.16E-09 |
|  | Deep | Archaea - Bacteria | -43.0642 | 44.05088 | 5291 | -0.9776 | 0.591081 |
|  |  | Archaea - Fungi | -922.301 | 48.92849 | 5291 | -18.85 | 1.20E-12 |
|  |  | Bacteria - Fungi | -879.236 | 38.43781 | 5291 | -22.8743 | 1.20E-12 |

Table S18. The number of nodes, separated by domain, characterised into four ecological roles based on their within-module connectivity and participation coefficient within the constructed networks.

| Network | Role | Kingdom | # of nodes |
| --- | --- | --- | --- |
| Surface | Peripherals | Archaea | 63 |
|  |  | Bacteria | 394 |
|  |  | Fungi | 136 |
|  | Connectors | Archaea | 64 |
|  |  | Bacteria | 523 |
|  |  | Fungi | 561 |
|  | Module hubs | Archaea | 4 |
|  |  | Bacteria | 15 |
|  |  | Fungi | 9 |
|  | Network hubs | Archaea | 0 |
|  |  | Bacteria | 0 |
|  |  | Fungi | 2 |
| Subsurface | Peripherals | Archaea | 126 |
|  |  | Bacteria | 360 |
|  |  | Fungi | 195 |
|  | Connectors | Archaea | 122 |
|  |  | Bacteria | 562 |
|  |  | Fungi | 203 |
|  | Module hubs | Archaea | 6 |
|  |  | Bacteria | 8 |
|  |  | Fungi | 10 |
|  | Network hubs | Archaea | 0 |
|  |  | Bacteria | 0 |
|  |  | Fungi | 0 |
| Deep | Peripherals | Archaea | 83 |
|  |  | Bacteria | 195 |
|  |  | Fungi | 210 |
|  | Connectors | Archaea | 279 |
|  |  | Bacteria | 821 |
|  |  | Fungi | 306 |
|  | Module hubs | Archaea | 5 |
|  |  | Bacteria | 8 |
|  |  | Fungi | 14 |
|  | Network hubs | Archaea | 0 |
|  |  | Bacteria | 0 |
|  |  | Fungi | 13 |

Table S19. The taxonomy of the identified network hubs, and their respective participation coefficient (P_i_) and within-module connectivity (Z_i_), in the surface and deep co-occurrence networks.

| Network | ASV | Taxonomy | P_i_ | Z_i_ |
| --- | --- | --- | --- | --- |
| Surface | 741 | Hypocreales | 0.6266 | 3.9165 |
|  | 2818 | Herpotrichiellaceae | 0.6583 | 3.2646 |
| Deep | 153 | *Pseudophaeosphaeria* | 0.7070 | 2.7110 |
|  | 208 | *Peroneutypa* | 0.7509 | 4.0090 |
|  | 280 | Pleosporales | 0.6263 | 3.1703 |
|  | 285 | *Kockovaella* | 0.6800 | 2.8389 |
|  | 290 | *Cytospora rhizophorae* | 0.6906 | 3.1698 |
|  | 500 | *Talaromyces* | 0.7116 | 2.5695 |
|  | 635 | *Scedosporium boydii* | 0.6848 | 3.2496 |
|  | 770 | Neocallimastigomycetes | 0.6517 | 2.8216 |
|  | 878 | Pleosporales | 0.6771 | 3.7427 |
|  | 884 | *Exophiala oligosperma* | 0.6861 | 4.2132 |
|  | 1235 | Microascales | 0.7096 | 2.8852 |
|  | 2305 | *Favolus acervatus* | 0.6704 | 2.5591 |
|  | 4709 | *Trichothecium* | 0.7648 | 3.6519 |

**References**

1. Ng MS, Soon N, Chang Y, Wainwright BJ. Bacterial and Fungal Co-Occurrence in the Nudibranch, Pteraeolidia semperi. Life. 2022;12:1988.

2. Ihrmark K, Bödeker ITM, Cruz-Martinez K, Friberg H, Kubartova A, Schenck J, et al. New primers to amplify the fungal ITS2 region – evaluation by 454-sequencing of artificial and natural communities. FEMS Microbiol Ecol. 2012;82:666–77.

3. White TJ, Bruns T, Lee S, Taylor J. Amplification and direct sequencing of fungal ribosomal RNA genes for phylogenetics. PCR Protoc Guide Methods Appl. 1990;18:315–22.

4. Caporaso JG, Lauber CL, Walters WA, Berg-Lyons D, Lozupone CA, Turnbaugh PJ, et al. Global patterns of 16S rRNA diversity at a depth of millions of sequences per sample. Proc Natl Acad Sci. 2011;108 supplement_1:4516–22.

5. Callahan BJ, McMurdie PJ, Rosen MJ, Han AW, Johnson AJA, Holmes SP. DADA2: High-resolution sample inference from Illumina amplicon data. Nat Methods. 2016;13:581–3.

6. Frøslev TG, Kjøller R, Bruun HH, Ejrnæs R, Brunbjerg AK, Pietroni C, et al. Algorithm for post-clustering curation of DNA amplicon data yields reliable biodiversity estimates. Nat Commun. 2017;8:1188.

7. Liber JA, Bonito G, Benucci GMN. CONSTAX2: improved taxonomic classification of environmental DNA markers. Bioinformatics. 2021;37:3941–3.

8. Davis NM, Proctor DM, Holmes SP, Relman DA, Callahan BJ. Simple statistical identification and removal of contaminant sequences in marker-gene and metagenomics data. Microbiome. 2018;6:226.
